# Lmx1b is required for the glutamatergic fates of a subset of spinal cord neurons

**DOI:** 10.1186/s13064-016-0070-1

**Published:** 2016-08-23

**Authors:** William C. Hilinski, Jonathan R. Bostrom, Samantha J. England, José L. Juárez-Morales, Sarah de Jager, Olivier Armant, Jessica Legradi, Uwe Strähle, Brian A. Link, Katharine E. Lewis

**Affiliations:** 1 Department of Biology, Syracuse University, 107 College Place, Syracuse, NY 13244 USA; 2 Department of Neuroscience and Physiology, SUNY Upstate Medical University, 505 Irving Avenue, Syracuse, NY 13210 USA; 3 Department of Cell Biology, Neurobiology and Anatomy, Medical College of Wisconsin, 8701 Watertown Plank Rd., Milwaukee, WI 53226 USA; 4 Department of Physiology, Development and Neuroscience, University of Cambridge, Downing Street, Cambridge, CB2 3DY UK; 5 Institute of Toxicology and Genetics, Karlsruhe Institute of Technology (KIT), Postfach 3640, 76021 Karlsruhe, Germany

**Keywords:** Spinal cord, Interneuron, Zebrafish, Lmx1b, Excitatory, Neurotransmitter, CNS, Transcription factor, V0v, dI5

## Abstract

**Background:**

Alterations in neurotransmitter phenotypes of specific neurons can cause imbalances in excitation and inhibition in the central nervous system (CNS), leading to diseases. Therefore, the correct specification and maintenance of neurotransmitter phenotypes is vital. As with other neuronal properties, neurotransmitter phenotypes are often specified and maintained by particular transcription factors. However, the specific molecular mechanisms and transcription factors that regulate neurotransmitter phenotypes remain largely unknown.

**Methods:**

In this paper we use single mutant, double mutant and transgenic zebrafish embryos to elucidate the functions of Lmx1ba and Lmx1bb in the regulation of spinal cord interneuron neurotransmitter phenotypes.

**Results:**

We demonstrate that *lmx1ba* and *lmx1bb* are both expressed in zebrafish spinal cord and that *lmx1bb* is expressed by both V0v cells and dI5 cells. Our functional analyses demonstrate that these transcription factors are not required for neurotransmitter fate specification at early stages of development, but that in embryos with at least two *lmx1ba* and/or *lmx1bb* mutant alleles there is a reduced number of excitatory (glutamatergic) spinal interneurons at later stages of development. In contrast, there is no change in the numbers of V0v or dI5 cells. These data suggest that *lmx1b-*expressing spinal neurons still form normally, but at least a subset of them lose, or do not form, their normal excitatory fates. As the reduction in glutamatergic cells is only seen at later stages of development, Lmx1b is probably required either for the maintenance of glutamatergic fates or to specify glutamatergic phenotypes of a subset of later forming neurons. Using double labeling experiments, we also show that at least some of the cells that lose their normal glutamatergic phenotype are V0v cells. Finally, we also establish that Evx1 and Evx2, two transcription factors that are required for V0v cells to acquire their excitatory neurotransmitter phenotype, are also required for *lmx1ba* and *lmx1bb* expression in these cells, suggesting that Lmx1ba and Lmx1bb act downstream of Evx1 and Evx2 in V0v cells.

**Conclusions:**

Lmx1ba and Lmx1bb function at least partially redundantly in the spinal cord and three functional *lmx1b* alleles are required in zebrafish for correct numbers of excitatory spinal interneurons at later developmental stages. Taken together, our data significantly enhance our understanding of how spinal cord neurotransmitter fates are regulated.

## Background

Neurons in the central nervous system (CNS) must specify and maintain several properties in order to integrate and function properly within neuronal circuitry [1]. One crucial neuronal characteristic that must be specified correctly and usually must be maintained (for some exceptions see [2]) is the neurotransmitter phenotype [1]. Failure to correctly specify or maintain neurotransmitter phenotypes can result in incorrect levels of excitatory or inhibitory neurotransmitter release and lead to diseases such as epilepsy, autism spectrum disorder, and Alzheimer’s [3–6].

Neurotransmitter phenotypes, like many other neuronal properties, are initially specified by transcription factors that individual neurons express as they start to differentiate [7–12]. These neurotransmitter phenotypes are then maintained either by these same transcription factors or by additional ones [7, 13–17]. However, in many cell types the transcription factors that specify and/or maintain neurotransmitter phenotypes are still unknown. This is a critical gap in our knowledge and one that we need to address in order to potentially develop better treatments for some of the aforementioned diseases and disorders.

In this paper, we investigate the functions of Lmx1b transcription factors in the zebrafish spinal cord. *Lmx1b* has been implicated in a variety of functions in different regions of the vertebrate CNS including cell migration, cell survival, as well as correct specification and/or maintenance of cell identity, neuronal connectivity and neurotransmitter phenotypes [18–25]. However, it remains unclear if *Lmx1b* is required for neurotransmitter specification and/or maintenance in the spinal cord.

Zebrafish have two *Lmx1b* ohnologs, *lmx1ba* and *lmx1bb,* that we show are probably expressed in overlapping spinal cord domains. Consistent with previous analyses in mouse, we show that *lmx1bb* is expressed by dI5 neurons, and for the first time in any animal, we show that V0v neurons (cells that form in the ventral part of the V0 domain [11, 12, 26–31]) also express *lmx1bb.* Both dI5 and V0v cells are glutamatergic [8, 11, 16, 31, 32] and consistent with this we demonstrate that the vast majority of *lmx1bb-*expressing cells are glutamatergic.

We also show in zebrafish *lmx1bb* homozygous mutants that glutamatergic neurons are correctly specified during early development but are reduced in number at later developmental time points. Interestingly, we see the same phenotype in *lmx1ba* homozygous mutants, *lmx1ba;lmx1bb* double mutants and *lmx1ba;lmx1bb* double heterozygous embryos suggesting that *lmx1ba* and *lmx1bb* act at least partially redundantly in a dose-dependent manner and that three functional *lmx1b* alleles are required for the specification or maintenance of correct numbers of spinal cord glutamatergic cells at later developmental stages. In contrast to the reduction in the number of glutamatergic neurons, there is no reduction in the numbers of V0v or dI5 cells in *lmx1bb* homozygous mutants and there is no increase in cell death. This suggests that *lmx1b*-expressing spinal neurons are still present in normal numbers at these later stages of development, but that fewer of them are glutamatergic. Interestingly, there is no increase in the number of inhibitory neurons, suggesting that the cells that are no longer excitatory do not become inhibitory. Finally, we demonstrate that *lmx1ba* and *lmx1bb* expression in V0v cells requires Evx1 and Evx2. In combination with a previous study that showed that Evx1 and Evx2 are required for V0v cells to become glutamatergic [11], this suggests that Lmx1ba and Lmx1bb act downstream of Evx1 and Evx2 either to maintain V0v glutamatergic fates or to specify the glutamatergic fates of a later-forming subset of V0v cells.

## Methods

### Zebrafish husbandry and fish lines

Zebrafish (*Danio rerio*) were maintained on a 14-h light/10-h dark cycle at 28.5 °C. Embryos were obtained from natural paired and/or grouped spawnings of wild-type (WT) (AB, TL or AB/TL hybrid) fish or identified heterozygous *lmx1bb*^*jj410*^*, lmx1ba*^*mw80*^*, evx1*^*i232*^*;evx2*^*sa140*^ or *smoothened*^*b641*^ mutant fish or *Tg*(*slc17a6:EGFP)* [formerly called *Tg(vGlut2a:EGFP)*] [33] or *Tg(evx1:EGFP)*^*SU1*^ [11] transgenic fish or *lmx1bb*^*jj410*^ crossed into the background of either *Tg(slc17a6b(vglut2a):loxP-DsRed-loxP-GFP)*^*nns14*^ [41, 42] or *Tg(evx1:EGFP)*^*SU1*^ fish respectively*.* Embryos were reared at 28.5 °C and staged by hours post fertilization (h) and/or days post fertilization (dpf). Most embryos were treated with 0.2 mM 1-phenyl 2-thiourea (PTU) at 24 h to inhibit melanogenesis [34–36].

The *evx1*^*i232*^, *evx2*^*sa140*^ and *lmx1bb*^*jj410*^ mutants have been previously described [11, 37–39]*.* All three of these mutations are single base pair changes that lead to premature stop codons before the homeobox. Therefore, if any of these RNAs are not degraded by nonsense mediated decay, the resulting proteins will lack the DNA binding domain. *lmx1ba*^*mw80*^ mutant zebrafish were generated using TALENs constructs that target the sequences TCAAGTAGACATGCTGGACG and TCCGCTCCTGTCCTGAACTG within the first exon of *lmx1ba.* Constructs were made using steps 1–38 outlined in [40]. To generate mRNA encoding the TALENs, approximately 5 μg of plasmid DNA was digested with ApoI and purified via the Invitrogen PureLink PCR Purification Kit (ThermoFisher, K310001). RNA was synthesized using the Ambion mMessage mMachine T7 kit (ThermoFisher, AM1344) with a poly(A) tail added from the Poly(A) Tailing Kit (Ambion, AM1350) and purified with the Megaclear Kit (Ambion, AM1908). 100 pg of RNA for each TALEN was co-injected into 1-cell WT embryos. The *lmx1ba*^*mw80*^ allele was recovered and identified as a single base pair deletion 20 bp into the coding sequence. This results in a frameshift after the first six amino acids and a premature stop codon 11 amino acids later. This stop codon is upstream of both the Lim and homeobox domains, suggesting that this allele is likely to be a complete loss of function.

### Genotyping

DNA for genotyping was isolated from both anesthetized adults and fixed embryos via fin biopsy or head dissections respectively. Fin biopsy and *evx1* and *evx2* genotyping of adults were performed as previously described [11, 37]. KASP assays, designed by LGC Genomics LLC, using DNA extracted from head dissections, were used to identify embryos carrying the *evx1*^*i232*^ and *evx2*^*sa140*^ mutations. These assays use allele-specific PCR primers which differentially bind fluorescent dyes that we quantified with a BioRad CFX96 real-time PCR machine to distinguish genotypes. The proprietary primers used are: Evx1_y32_i232 and Evx2_sa140.

Heads of fixed embryos were dissected in 80 % glycerol/20 % phosphate-buffered saline (PBS) with insect pins. Embryo trunks were stored in 70 % glycerol/30 % PBS at 4 °C for later analysis. DNA was extracted via the HotSHOT method [41] using 20 μL of NaOH and 2 μL of Tris-HCl (pH-7.5).

The *lmx1ba*^*mw80*^ and *lmx1bb*^*jj410*^ alleles were identified by restriction enzyme digestion assays as both of these mutations disrupt endogenous restriction enzyme sites. For *lmx1ba*^*mw80*^, a 540 bp amplicon encompassing the mutation site was generated with the following primers:

Forward GATCCTCAAGAGGAGCTCATACACA and Reverse CATGCACATTTAACTATGATCTGAGCCGTG.

This amplicon was digested with MluCI to yield 311 bp and 142 bp and 87 bp (WT), 453 bp and 87 bp (homozygous mutant), or 453 bp and 311 bp and 142 bp and 87 bp (heterozygous mutant) products. Similarly, for *lmx1bb*^*jj410*^, a 264 bp amplicon encompassing the mutation site was generated with the following primers: Forward GAAGGCTCGTCTCTGCTGTGTGGTG and Reverse CGTTATGGATGCGCTGAGACTGAATACC. This amplicon was digested with BfaI to yield 211 bp and 53 bp (WT), 264 bp (homozygous mutant), or 264 bp and 211 bp and 53 bp (heterozygous mutant) products.

### Expression profiling V0v neurons & microarray design

To identify transcription factors expressed by V0v neurons, V0v spinal neurons, all spinal cord neurons and all cells within the trunk were isolated from live transgenic zebrafish embryos at 27 h using fluorescence activated cell-sorting (FACS). Prior to FACS, embryos were prim-staged, de-yolked, dissected and dissociated as in [42, 43]. Heads and tails were removed from all samples to ensure that only trunk or spinal cord cells were collected. Trunk samples correspond to FAC-sorted trunk cells (spinal cord and other tissues). All neuron samples are EGFP-positive cells from *Tg(elav13:EGFP)* trunks [44]. V0v neurons are EGFP-positive cells from *Tg(evx1:EGFP)*^*SU1*^ trunks [11]. Total RNA was extracted using an RNeasy Micro Kit (Qiagen, 74004). The quality of RNA was assessed via an Agilent 2100 Bioanalyser (RNA 6000 Pico Kit, Agilent, 5067–1513) before being converted to fluorescently-labeled cDNA (Ovation Pico WTA System V2, Pico, 3302) and hybridized to a custom-designed Agilent microarray (Agilent #027382). Data pre-processing and normalization was performed using Bioconductor software (https://www.bioconductor.org/). A three-class ANOVA analysis was performed using GEPAS software [45, 46]. Relative expression levels were subjected to a Z-transformation normalization and are presented as Z scores where mean = 0 and standard deviation = +1 (red) to -1 (blue) [47–49]. All reported statistics were corrected for multiple testing [50].

To generate the custom-designed Agilent microarray (Agilent #027382) we first performed comprehensive bioinformatic searches for proteins that contain at least one of the 483 InterPro domains identified in [51] as being specific to transcriptional regulators. These domains comprise three functional classes: DNA binding, chromatin remodeling and general transcription machinery. We identified 3192 potential transcription factors. 2644 of these proteins were identified in Zv8 (Ensembl release 54) of the zebrafish genome and a further 548 non-overlapping transcription factors were identified in the zebrafish Unigene dataset (release 117). Our custom arrays contain 33784 probes corresponding to eight distinct 60-mer probes for each of the transcripts associated with these 3192 proteins. We also included 170 housekeeping genes (five copies of eight probes each), 23 positive controls, such as neurotransmitter markers (two copies of eight probes each) and 49 negative controls (Arabidopsis sequences; multiple copies of eight probes each) on the arrays. Four biological replicates were performed per sample type. Microarray data are deposited at NCBI GEO entry number GSE83723.

### *in situ* hybridization

Embryos were fixed in 4 % paraformaldehyde and single and double *in situ* hybridization experiments were performed as previously described [52, 53]. Probes for *in situ* hybridization experiments were prepared using the following templates: *evx1* [30]*, evx2* [29]*, lbx1a* [54] and *lmx1ba* [24]*.* A probe for *lmx1bb* was generated from cDNA as previously described [11, 43] with the following primers: forward CTGGATATCAAGCCGGAGAA; reverse AATTAACCCTCACTAAAGGGATCCGAACATCACATTTCAACA. The *lmx1bb* probe sequence was selected to avoid cross-hybridization with *lmx1ba* and other *lmx1* family members.

To try and improve signal strength of the *lmx1ba* probe, we also hydrolyzed the full length *lmx1ba* probe described above [24] to approximately 200 bp fragments as outlined in [55] and tested two additional *lmx1ba* probes. The second probe was synthesized from a plasmid containing the last 584 bp of the coding sequence of *lmx1ba.* The third probe, which recognizes the 3’ coding sequence and UTR of *lmx1ba,* was generated from cDNA, as previously described [11, 43], with the following primers: forward CGCATGCGTTGGTATCTATG; reverse AATTAACCCTCACTAAAGGGAAAGCATCCTCCACAATGTCC. As these probes did not improve the signal quality when compared to the first probe described above [24], results from these *in situ* hybridization experiments are not included in this paper.

To determine neurotransmitter phenotypes, we used *in situ* probes for genes that function as transporters of neurotransmitters or that synthesize specific neurotransmitters as these are some of the most specific molecular markers of these cell fates (e.g. see [56] and references therein). A mixture of probes to *slc17a6a* and *slc17a6b*, which encode glutamate transporters, was used to label glutamatergic neurons [56, 57]. To label inhibitory cells we used *slc32a1*, which encodes a vesicular inhibitory amino acid transporter [33]. To label glycinergic cells a mixture of probes (*glyt2a* and *glyt2b*) for the gene *slc6a5* were used [56, 57]. The *slc6a5* gene encodes for a glycine transporter necessary for glycine reuptake and transport across the plasma membrane. GABAergic neurons were labeled by a mixture of probes to *gad1b* and *gad2* genes (probes previously called *gad67a, gad67b* and *gad65*) [56, 57]. The *gad1b* and *gad2* genes encode for glutamic acid decarboxylases, which are necessary for the synthesis of GABA from glutamate.

### Immunohistochemistry

Embryos were fixed in 4 % paraformaldehyde and stored in PBS with 0.1 % tween20. To permeabilize embryos they were treated with acetone at -20 °C for 30 min (36 h or younger), 1 h (48 h) or 3 h (7 dpf) and then processed as previously described [11]. Primary antibodies were: mouse anti-GFP (Roche Applied Science, 11814460001, 1:500), rabbit anti-DsRed (Clontech, 632496, 1:200) or rabbit anti-activated caspase-3 (Fisher Scientific/BD, BDB559565, 1:500). Secondary antibodies were: Alexa Fluor 568 goat anti-rabbit (Molecular Probes, A11036, 1:500), Alexa Fluor 488 goat anti-mouse (Molecular Probes, A11029, 1:500) or Alexa Fluor 488 goat anti-rabbit (Molecular Probes, A11034, 1:500).

### Double stains

Both double *in situ* hybridization and immunohistochemistry plus *in situ* hybridization double labeling experiments were performed as in [52].

### Acridine orange treatment

A stock acridine orange base (Sigma-Aldrich, 235474) solution of 2.5 mg/mL in dimethyl sulfoxide (DMSO) was made and stored at -20 °C until used. At 24 h, 36 h and 48 h acridine orange stock solution was added to embryo medium (5 mM NaCl, 0.17 mM KCl, 0.33 mM CaCl_2_ · 2H_2_O and 0.33 mM MgSO_4_ · 7H_2_O in water) to make a final concentration of 5 μg/mL. Embryos were bathed in the acridine orange / embryo medium solution in the dark at 28.5 °C for 28 min. Embryos were then washed five times in embryo medium for 5 min each and analyzed using fluorescent microscopy on a Zeiss Axio Imager M1 compound microscope and Olympus SZX16 dissecting microscope.

### Imaging

Embryos were mounted in 70 % glycerol, 30 % PBS and differential interference contrast (DIC) pictures were taken using an AxioCam MRc5 camera mounted on a Zeiss Axio Imager M1 compound microscope. Embryos from acridine orange experiments and anti-activated caspase-3 experiments were mounted in 2 % 1,4-diazabicyclo[2.2.2] octane (DABCO) and imaged in the same way. Zeiss LSM 710 and LSM 780 confocal microscopes were used to image embryos mounted in DABCO from fluorescent *in situ* hybridization and immunohistochemistry experiments. Images were processed using Adobe Photoshop software (Adobe, Inc), GNU Image Manipulations Program (GIMP 2.6.10, http://gimp.org) and Image J software (Abramoff et al. [58]). In some cases, different focal planes were merged to show labeled cells at different medial-lateral positions in the spinal cord.

### Cell counts and statistics

For acridine orange staining and activated caspase-3 immunohistochemistry experiments, cells were counted along both sides of the entire rostral-caudal axis of the spinal cord. For all other experiments, we identified somites 6–10 in each embryo and counted the number of labeled cells in that stretch of the spinal cord. In all cases, embryos were mounted laterally with the somite boundaries on each side of the embryo exactly aligned and the apex of the somite over the middle of the notochord. This ensures that the spinal cord is straight along its dorsal-ventral axis and that cells in the same dorsal/ventral position on opposite sides of the spinal cord will be directly above and below each other. Cell counts for fluorescently-labeled cells were performed by analyzing all focal planes in a confocal stack of the appropriate region(s) of the spinal cord. Labeled cells in embryos analyzed by DIC were counted while examining embryos on the Zeiss Axio Imager M1 compound microscope. We adjusted the focal plane as we examined the embryo to count cells at all medial/lateral positions (both sides of the spinal cord; also see [7, 11, 52, 59]). Values are reported as the mean +/- the standard error of the mean. Results were analyzed using the student’s t*-*test.

## Results

### *lmx1ba* and *lmx1bb* are expressed by zebrafish dI5 and V0v neurons

**Fig. 1:**
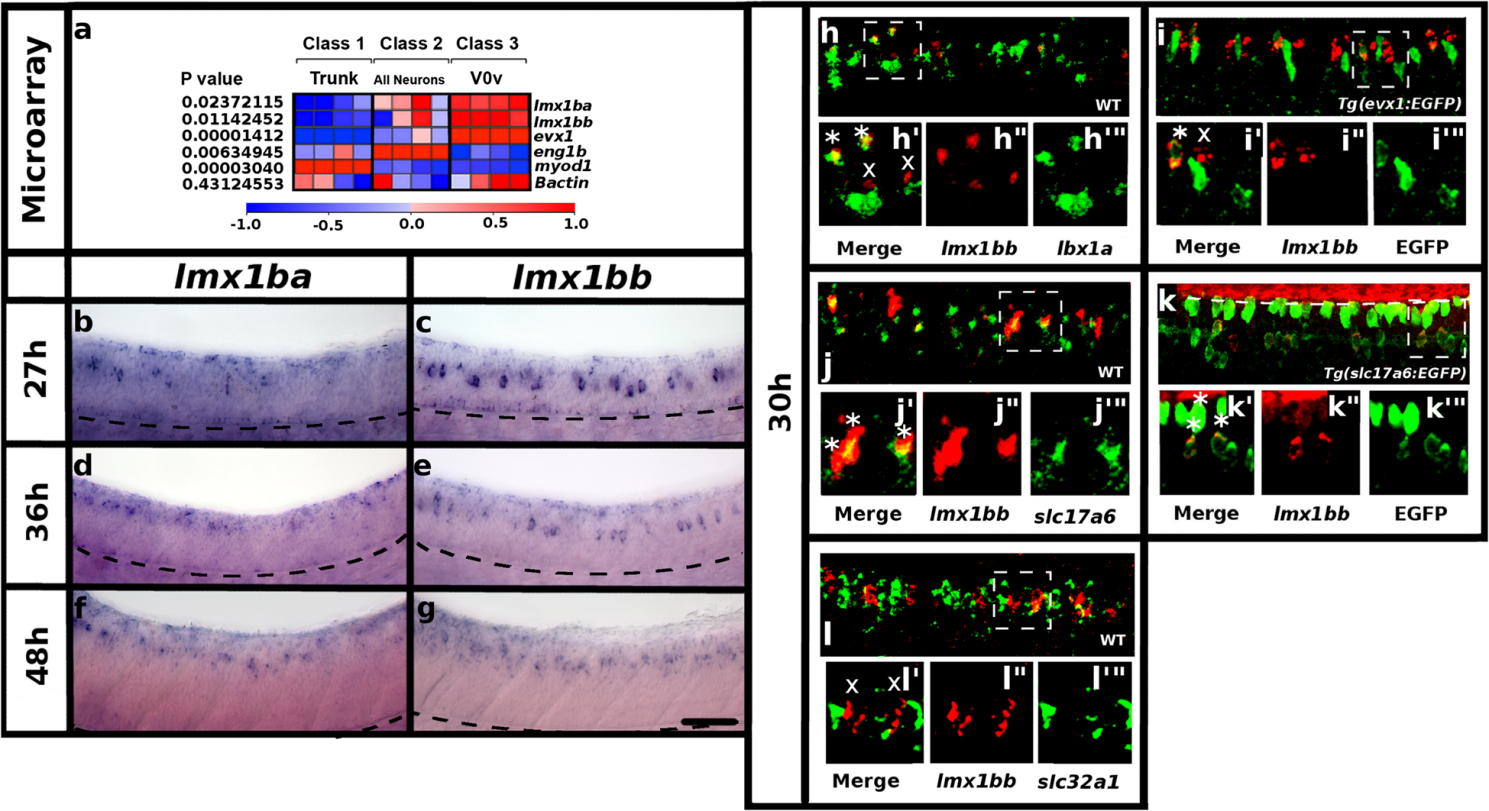
*lmx1b* expression in zebrafish spinal cord. **a** Three-class ANOVA comparison of V0v cells (class 3), trunk cells (class 1) and all post-mitotic neurons (class 2). *p* values test hypothesis that there is no differential expression among the 3 classes. Columns represent individual microarray experiments. Rows indicate relative expression levels as normalized data, subjected to a Z-transformation where mean = 0 and standard deviation = 1, where red = normalized expression value of +1 and blue = normalized expression value of -1 (see Methods for more details). *lmx1ba* and *lmx1bb* are expressed by V0v neurons. Positive control *evx1* is also expressed by V0v neurons. Negative controls *eng1b* and *myod1* are expressed by other neurons (V1 cells) and trunk cells respectively. *βactin* is a housekeeping gene that is expressed by all populations. **b**-**l** Lateral views of zebrafish spinal cord at 27 h (**b** and **c**), 30 h (**h**-**l**), 36 h (**d** and **e**) and 48 h (**f** and **g**). Anterior left, dorsal top. *in situ* hybridization for *lmx1ba* (**b**, **d** and **f**) and *lmx1bb* (**c**, **e** and **g**). Black dashed line (**b**-**g**) is just below ventral limit of spinal cord, floor plate is right above this, in the most ventral part of the spinal cord, roof plate is the most dorsal part of the spinal cord. Double *in situ* hybridization for *lmx1bb* (red) and *lbx1a* (green) in WT embryo, merged view (**h**) and magnified single confocal plane of white dotted box region (**h’**-**h’”**). *in situ* hybridization for *lmx1bb* (red) and EGFP immunohistochemistry (green) in *Tg(evx1:EGFP)* ^*SU1*^ embryo, merged view (**i**) and magnified single confocal plane of white dotted box region (**i’**-**i’”**). Double *in situ* hybridization for *lmx1bb* (red) and *slc17a6* (green) in WT embryo, merged view (**j**) and magnified single confocal plane of white dotted box region (**j’**-**j’”**). *in situ* hybridization for *lmx1bb* (red) and EGFP immunohistochemistry (green) in *Tg*(*slc17a6:EGFP)* embryo, merged image (**k**) and magnified single confocal plane of white dotted box region (**k’**-**k’”**). White dashed line (**k**) marks the dorsal limit of the spinal cord. Red staining above the dashed line is outside the spinal cord. Double *in situ* hybridization for *lmx1bb* (red) and *slc32a1* (green) in WT embryo, merged image (**l**) and magnified single confocal plane of white dotted box region (**l’**-**l’”**). In all cases (**h**-**l**) * indicates co-labeled cell, x indicates single labeled *lmx1bb*-expressing cell. In all cases, at least two independent double-labeling experiments were conducted (**h**-**l**). Results were similar for each replicate. Numbers of single and double-labeled cells and number of embryos counted are provided in Tables 1 and 2. Scale bar = 50 μm (**b**-**g**), 70 μm (**h**-**l**) and 20 μm (**h’**-**l’”**)

To identify transcription factors that may play a role in V0v neuron specification and/or maintenance, we expression-profiled V0v neurons and compared them to all post-mitotic neurons and all trunk cells (see methods; NCBI GEO GSE83723; [43]). These analyses identified *lmx1ba* and *lmx1bb,* zebrafish ohnologs of Lmx1b (Fig. 1a), as two transcription factor genes potentially expressed in V0v neurons. Prior to this study, the only report of *lmx1b* expression in the zebrafish spinal cord established that *lmx1bb* is expressed in at least some rostral spinal neurons at 24 h [24]. However, it was unclear if *lmx1bb* expression was restricted to the rostral spinal cord and these earlier studies did not detect *lmx1ba* expression in the spinal cord [24]. Therefore, to further confirm our microarray data, we examined the spinal cord expression of *lmx1ba* and *lmx1bb* in more detail (Fig. 1).

At 27 h, *lmx1ba* is expressed in a narrow dorsal-ventral domain by interneurons in the most rostral region of the spinal cord, as well as in cells of the roof plate and floor plate (Fig. 1b). As development progresses, additional interneurons start to express *lmx1ba* and expression extends more caudally in the spinal cord (Fig. 1b, d and f, Table 1). By 48 h, *lmx1ba* expression is no longer detected in the floor plate but is still present in the roof plate and interneurons (Fig. 1f).

**Table 1:** *lmx1ba* and *lmx1bb* are expressed in zebrafish spinal cord

	*lmx1ba-*expressing cells				*lmx1bb-*expressing cells			
	27 h	30 h	36 h	48 h	27 h	30 h	36 h	48 h
Mean	3.5	8.6	11.8	22.5	31.1	37.6	57	80.4
SEM	1	1.8	0.5	2.4	1.4	1.7	1.3	1.9
n	4	5	4	4	11	15	4	5

Mean number of interneurons (roof and floor plate expression is excluded) expressing *lmx1ba* (columns 2–5) or *lmx1bb* (columns 6–9) at 27, 30, 36 and 48 h in the spinal cord region adjacent to somites 6–10. SEM indicates the standard error of the mean for each time point analyzed. n is the number of embryos analyzed. The *lmx1ba* probe is very weak so it is possible that we only detected the most strongly-expressing spinal cord cells

In contrast, at 27 h, *lmx1bb* spinal cord expression already extends along the entire rostral-caudal axis in a narrow dorsal-ventral domain (Fig. 1c). Like *lmx1ba, lmx1bb* is also expressed in the roof plate and floor plate at this stage. As development progresses, more spinal cord neurons express *lmx1bb* and roof plate expression becomes more prominent while floor plate expression is lost by 36 h (Fig. 1c, e and g; Table 1). By 48 h, *lmx1ba* and *lmx1bb* are expressed in presumably overlapping domains, although, as all *lmx1ba in situ* probes tested produced very weak staining (see methods), it was not possible to confirm this with co-labeling experiments.

To determine the specific spinal cell types that express *lmx1bb* we performed double-labeling experiments. In mouse, Lmx1b is expressed by dI5 neurons that also express Lbx1 [18, 32, 60–64]. To test if this is also the case in zebrafish, we performed a double *in situ* hybridization for *lmx1bb* and *lbx1a.* At 30 h we found that approximately 45 % of *lmx1bb-*expressing cells co-express *lbx1a* (Fig. 1h; Table 2)*.* These results suggest that only a subset of *lmx1bb-*expressing neurons are dI5 neurons. In mouse three populations of neurons (dI4, dI5 and dI6) express the transcription factor Lbx1 but only the excitatory dI5 neurons express Lmx1b while inhibitory dI4 and dI6 cells do not [18, 32, 60–64]. Similarly, we find that in the zebrafish spinal cord only 33 % of *lbx1a-*expressing cells co-express *lmx1bb* (Fig. 1h; Table 2).

**Table 2:** Co-expression of other genes with *lmx1bb*

*lmx1bb* + *Tg(slc17a6:EGFP)* double labeling experiments			
30 h	*lmx1bb*	*Tg(slc17a6:EGFP)*	co-labeled
Mean	30	105.7	21
SEM	3	9.7	2.2
n	7	7	7
%	70 %	20 %	n/a
*lmx1bb* + *slc17a6* double labeling experiments			
30 h	*lmx1bb*	*slc17a6*	co-labeled
Mean	32.5	99.8	25.8
SEM	1.1	4.4	1.3
n	4	4	4
%	79 %	26 %	n/a
*lmx1bb* + *slc32a1* double labeling experiments			
30 h	*lmx1bb*	*slc32a1*	co-labeled
Mean	28.3	142.7	3
SEM	1.2	2.4	0.6
n	6	6	6
%	10 %	2 %	n/a
*lmx1bb* + *Tg(evx1:EGFP)* double labeling experiments			
30 h	*lmx1bb*	*Tg(evx1:EGFP)*	co-labeled
Mean	36	70.5	13.5
SEM	2.1	2.4	1.7
n	6	6	6
%	38 %	19 %	n/a
*lmx1bb* + *lbx1a* double labeling experiments			
30 h	*lmx1bb*	*lbx1a*	co-labeled
Mean	29.4	40	13.3
SEM	1.7	2.7	1
n	7	7	7
%	45 %	33 %	n/a

Number of cells detected in co-labeling experiments. Mean number of cells that express *lmx1bb* (column 2), or gene being assessed for co-expression (column 3) in the spinal cord region adjacent to somites 6–10. Column 4 shows the number of these cells that have co-localized expression. SEM values indicate the standard error of the mean for each value. n values are the number of embryos counted and averaged for each result shown here. % values indicate the percentage of *lmx1bb*-expressing cells that have co-localized expression with other genes being assessed (column 2) or the % of cells that expressed other genes that have co-localized expression with *lmx1bb* (column 3)

As mentioned above, our expression profiling of V0v neurons suggested that zebrafish *lmx1b* genes may also be expressed by these cells (Fig. 1a). To confirm these results we performed EGFP immunohistochemistry and *lmx1bbin situ* hybridization in *Tg(evx1:EGFP)*^*SU1*^ embryos that express EGFP in V0v neurons [11]. These experiments showed that at 30 h at least 38 % of *lmx1bb-*expressing neurons are V0v neurons (Fig. 1i; Table 2).

Both V0v cells and dI5 cells are glutamatergic [8, 11, 16, 33, 34]. Moreover, *Lmx1b*-expressing neurons are glutamatergic in the amniote spinal cord [8, 16, 32]. Therefore, to further confirm the identity of zebrafish *lmx1bb-*expressing spinal neurons we performed double-labeling experiments. Double *in situ* hybridization for *lmx1bb* and glutamatergic markers *slc17a6a* + *slc17a6b* (a mixture of probes for both genes, referred to here as *slc17a6*; see methods), showed that at 30 h at least 79 % of *lmx1bb-*expressing cells co-express *slc17a6* (Fig. 1j; Table 2). To further confirm that most *lmx1bb-*expressing neurons are glutamatergic, we also performed double staining for EGFP and *lmx1bb* in 30 h *Tg(slc17a6:EGFP)* embryos in which many glutamatergic neurons express EGFP [33, 65–67]. In these embryos, we found that approximately 70 % of *lmx1bb-*expressing neurons also express EGFP (Fig. 1k; Table 2). In contrast, double *in situ* hybridization with *lmx1bb* and *slc32a1,* which labels all spinal cord inhibitory neurons [33, 68], revealed that only 10 % of *lmx1bb* neurons are inhibitory (Fig. 1l; Table 2). Taken together, these data suggest that the vast majority of zebrafish *lmx1bb-*expressing cells are glutamatergic and that these glutamatergic cells correspond to dI5 and V0v neurons.

### *lmx1bb* is required for glutamatergic neurotransmitter phenotypes at later developmental stages but does not repress inhibitory neurotransmitter phenotypes

**Fig. 2:**
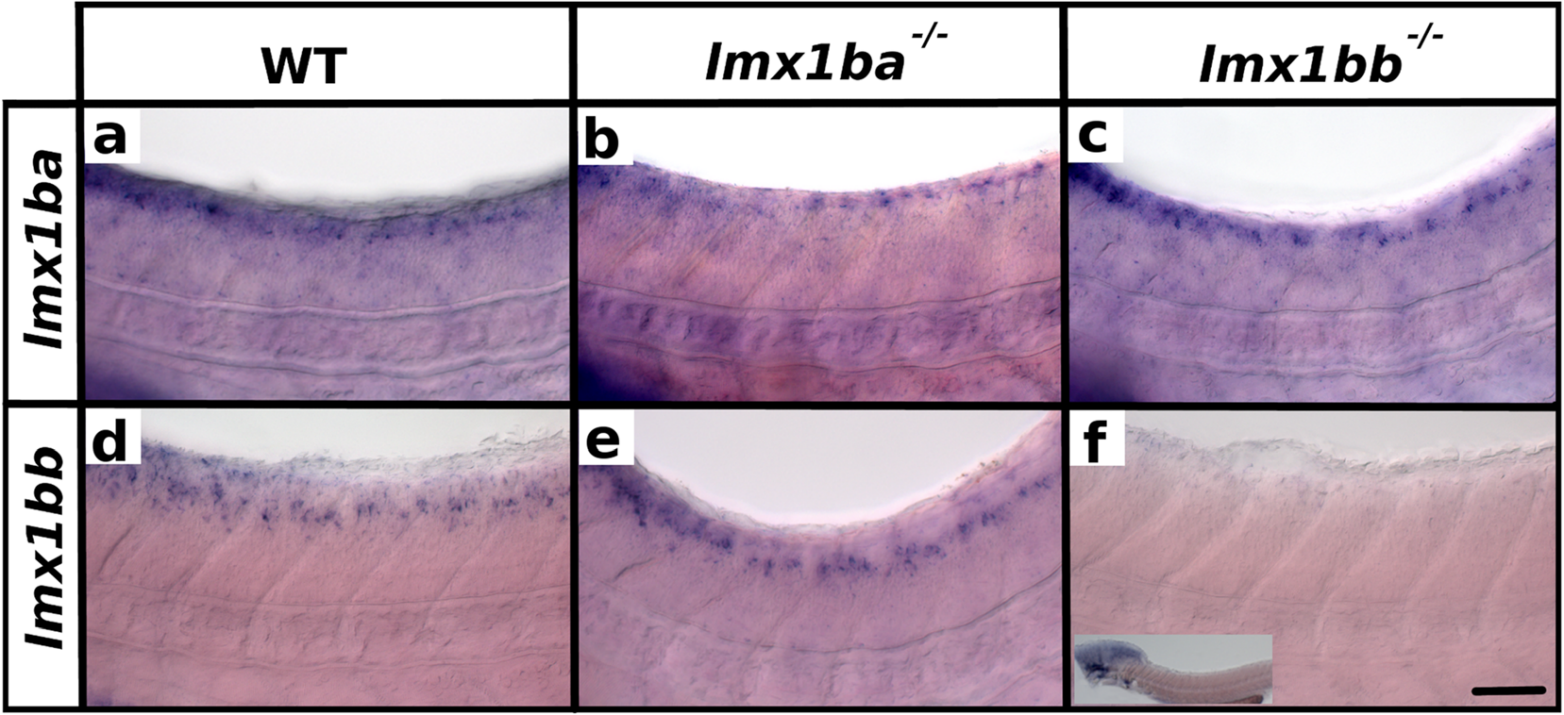
Expression of *lmx1b* RNAs in *lmx1b* mutants. Lateral view of zebrafish spinal cord at 48 h (**a**-**f**). Anterior left, dorsal top. *in situ* hybridization of *lmx1ba* (**a**-**c**) or *lmx1bb* (**d**-**f**) in WT (**a** and **d**), *lmx1ba* mutant (**b** and **e**) and *lmx1bb* mutant (**c** and **f**). Lower magnification insert in (**f**) shows expression remaining in hindbrain region. The rest of the head was removed for genotyping. One *in situ* hybridization of at least 40 embryos was conducted for each of **b** and **e**. Two independent *in situ* hybridizations of at least 50 embryos each were conducted for **a**, **c**, **d** and **f**. In these cases, results were the same for each replicate experiment. At least three genotyped mutant and wild-type embryos were analyzed in detail for each experiment. Scale bar = 50 μm

To investigate the functions of *lmx1ba* and *lmx1bb* in the zebrafish spinal cord we used mutations in each of these genes (see methods). We consider that both of these mutant alleles are likely to cause a complete loss of function as they result in premature stop codons before the homeobox (*lmx1bb*) or before both the homeobox and the lim domains (*lmx1ba*) (see methods). In fact, if the mutated *lmx1ba* RNA is translated, it would consist of only six amino acids of WT sequence followed by 11 altered amino acids. To test if the RNAs are degraded by nonsense mediated decay, we performed *in situ* hybridization for each gene in the respective mutant. For *lmx1ba,* we do not see any obvious changes in *lmx1ba* RNA (Fig. 2b). In contrast, we see a loss of *lmx1bb* RNA in the spinal cord of *lmx1bb* homozygous mutants (Fig. 2f), although some, potentially weaker than normal, expression remains in other regions of the embryo. This suggests that at least Lmx1bb function is completely lost from the spinal cord.

**Fig. 3:**
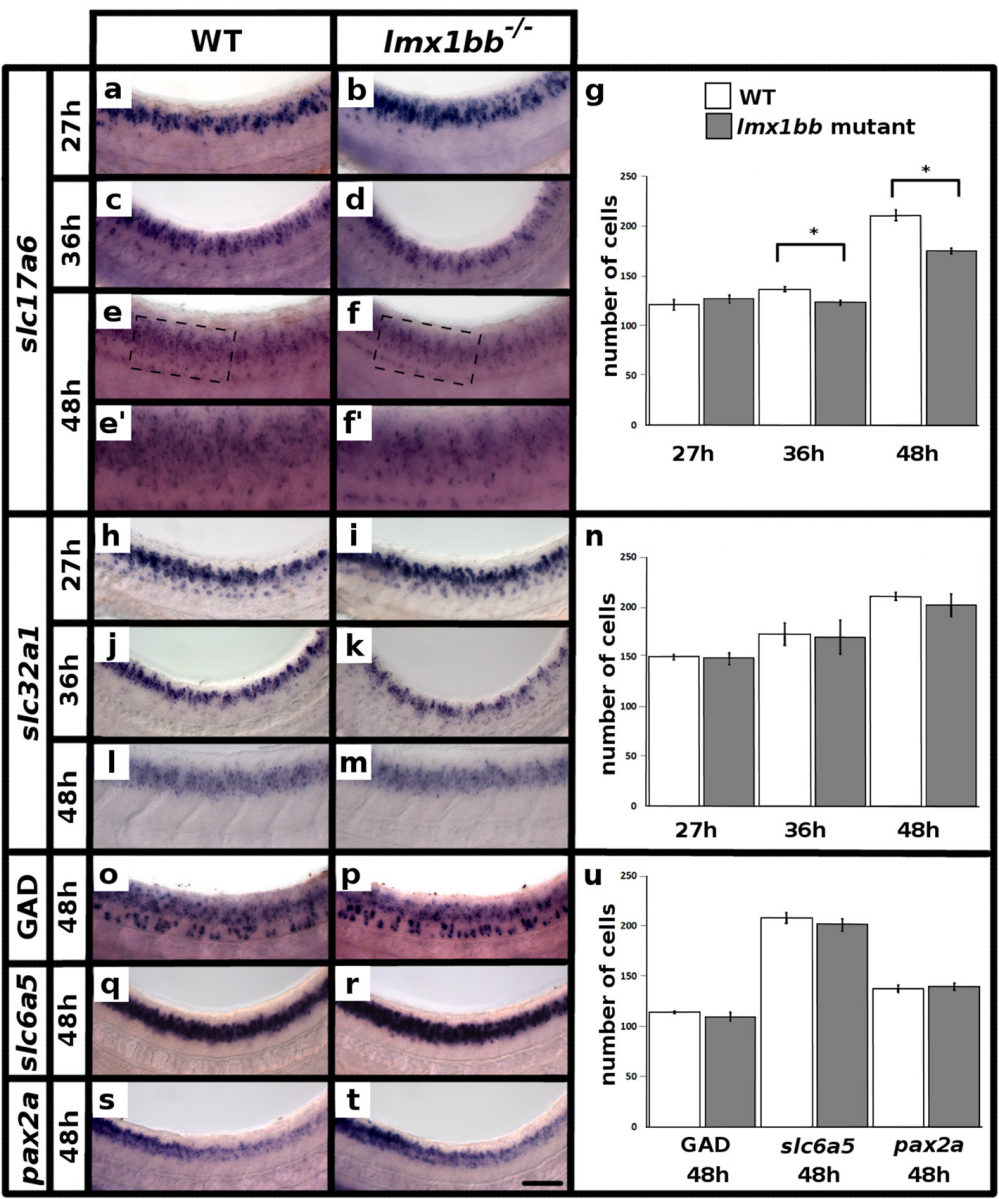
*lmx1bb* is required for glutamatergic phenotypes at later developmental stages but does not repress inhibitory phenotypes. Lateral view of zebrafish spinal cord at 27 h (**a**, **b**, **h** and **i**), 36 h (**c**, **d**, **j** and **k**) and 48 h (**e**-**f’**, **l**, **m** and **o**-**t**), anterior left, dorsal top. *in situ* hybridization for *slc17a6a* + *slc17a6b* (*slc17a6*) (**a**-**f’**), *slc32a1* (**h**-**m**), *gad1b* + *gad2* (GAD) (**o**, **p**), *slc6a5* (**q** and **r**) and *pax2a* (**s** and **t**). (**e’** and **f’**) are magnified views of black dashed box region in (**e** and **f**) respectively. Mean number of cells (y-axis) expressing markers *slc17a6* (**g**), *slc32a1* (**n**) and GAD*, slc6a5* or *pax2a* at 48 h (**u**) in spinal cord region adjacent to somites 6–10 in WT embryos (white) and *lmx1bb* homozygous mutants (grey) (x-axis). Statistically significant (*p* < 0.05) comparisons are indicated with square brackets and stars. Error bars indicate standard error of the mean. Two independent experiments were conducted for all *slc17a6* and *slc32a1* experiments (**a**-**m**). Cells count results were similar for each replicate. One experiment was conducted for (**o**-**t**). Cell count data presented here (**g**, **n** and **u**) are average values for 4 to 17 embryos from the same *in situ* hybridization experiment. Precise numbers of embryos counted and *p* values are provided in Tables 3 and 4. Scale bar = 50 μm (**a**-**f**, **h**-**m** and **o**-**t**) and 25 μm (**e’** and **f’**)

Since we see a loss of *lmx1bb* spinal cord expression in *lmx1bb* mutants and *lmx1bb* is expressed by more spinal interneurons at an earlier developmental time point than *lmx1ba*, we first examined the function of *lmx1bb*. As *lmx1bb* is expressed predominantly by glutamatergic neurons in the spinal cord, we assessed the expression of the glutamatergic marker *slc17a6* at 27, 36, and 48 h [18, 32]. At 27 h there was no statistically significant difference in the number of glutamatergic neurons in the spinal cord (*p* = 0.41, Fig. 3a, b and g; Table 3). However, at 36 h there was a statistically significant reduction in the number of glutamatergic neurons in *lmx1bb* mutants compared to WT siblings (*p* < 0.001, Fig. 3c, d and g; Table 3) and this reduction became more pronounced by 48 h (*p* < 0.001, Fig. 3e-g; Table 3). Taken together, these results suggest that *lmx1bb* is required either to maintain the glutamatergic phenotype of a subset of excitatory spinal neurons or to specify the glutamatergic phenotype of a later-forming subset of neurons.

**Table 3:** Lmx1bb is required for excitatory and not inhibitory neurotransmitter phenotypes

		27 h		36 h		48 h	
Marker		WT	*lmx1bb* ^*-/-*^	WT	*lmx1bb* ^*-/-*^	WT	*lmx1bb* ^*-/-*^
*slc17a6*	Mean	121.6	127.2	137.2	123.6	211	175
	SEM	5.3	4.1	2.5	2.4	5.5	2.9
	n	10	10	13	17	10	13
	*p* value	0.411		**<0.001**		**<0.001**	
*slc32a1*	Mean	149.7	148	173.2	169.7	210.5	202
	SEM	2.7	5.8	10.9	17.1	4	10.8
	n	10	6	14	7	12	6
	*p* value	0.77		0.85		0.48	

Mean number of *slc17a6a* + *slc17a6b* (*slc17a6*) or *slc32a1-*expressing cells counted in the spinal cord region adjacent to somites 6–10 in 27 h, 36 h and 48 h embryos. SEM is the standard error of the mean. n is the number of embryos analyzed for each data set. *p* value is from a student’s paired t-test comparing WT and *lmx1bb* mutant embryos. Statistically significant *p* values are indicated in bold

To determine if these neurons switch their neurotransmitter phenotype in *lmx1bb* mutants we examined markers of inhibitory cells. We did not detect any statistically significant changes in the number of inhibitory neurons expressing *slc32a1* at 27 h, 36 h, or 48 h in *lmx1bb* mutant embryos (*p* = 0.77, 0.85 and 0.48 respectively; Fig. 3h-n; Table 3). To further confirm these results, we examined the expression at 48 h of *gad1b* + *gad2* (a mixture of probes for both genes, referred to here as GADs)*,* which specifically label GABAergic neurons [69–71], and *slc6a5,* which specifically labels glycinergic neurons [72–75]. Consistent with the *slc32a1* findings, we also saw no statistically significant change in the number of GABAergic or glycinergic spinal neurons in *lmx1bb* mutants when compared to WT siblings (*p* = 0.54 and 0.38 respectively; Fig. 3o-r and u; Table 4). We also examined expression of *pax2a*, which encodes for a transcription factor that is required for the inhibitory neurotransmitter phenotypes of several classes of spinal interneurons [7, 9, 10, 13, 17]. Consistent with our other results, *pax2a* expression was unchanged in *lmx1bb* mutants (*p* = 0.7; Fig. 3s-u; Table 4). Taken together, these results suggest that there is no change in the number of inhibitory spinal neurons in *lmx1bb* mutants.

**Table 4:** Expression of genes in WT and *lmx1bb* mutant embryos

Marker	48 h	WT	*lmx1bb* ^*-/-*^
GAD	Mean	114.2	109.5
	SEM	1.2	4.5
	n	5	4
	*p* value	0.54	
*slc6a5*	Mean	208.3	201.2
	SEM	4.9	6.2
	n	6	6
	*p* value	0.38	
*pax2a*	Mean	138	140
	SEM	3.4	3.4
	n	7	5
	*p* value	0.7	
*lbx1a*	Mean	96.5	96.1
	SEM	3.6	2.4
	n	8	8
	*p* value	0.93	
*evx*	Mean	90.9	92.1
	SEM	1.9	3.9
	n	10	6
	*p* value	0.78	
*Tg(evx1:EGFP)* ^*SU1*^	Mean	94.9	96.3
	SEM	2.6	0.94
	n	8	8
	*p* value	0.63	

Mean number of *gad1b* + *gad2* (GAD), *slc6a5, pax2a, lbx1a, evx1* + *evx2* (*evx*) or *Tg(evx1:EGFP)* ^*SU1*^ *-*expressing cells in the spinal cord region adjacent to somites 6–10 in 48 h embryos. SEM is the standard error of the mean. n is the number of embryos analyzed for each data set. *p* value is from a student’s paired t-test comparing WT and *lmx1bb* mutant embryos

### *lmx1ba* and *lmx1bb* single mutants and *lmx1ba;lmx1bb* double mutants have the same spinal cord phenotype

As shown above (Fig. 1d-g), *lmx1ba* and *lmx1bb* are expressed in potentially overlapping domains within the zebrafish spinal cord during the developmental time points that we detected neurotransmitter phenotypes in *lmx1bb* mutants. This suggested that these two ohnologs might function redundantly in the spinal cord. Therefore, we examined spinal cord neurotransmitter phenotypes in *lmx1ba* single and *lmx1ba;lmx1bb* double mutants.

**Fig. 4:**
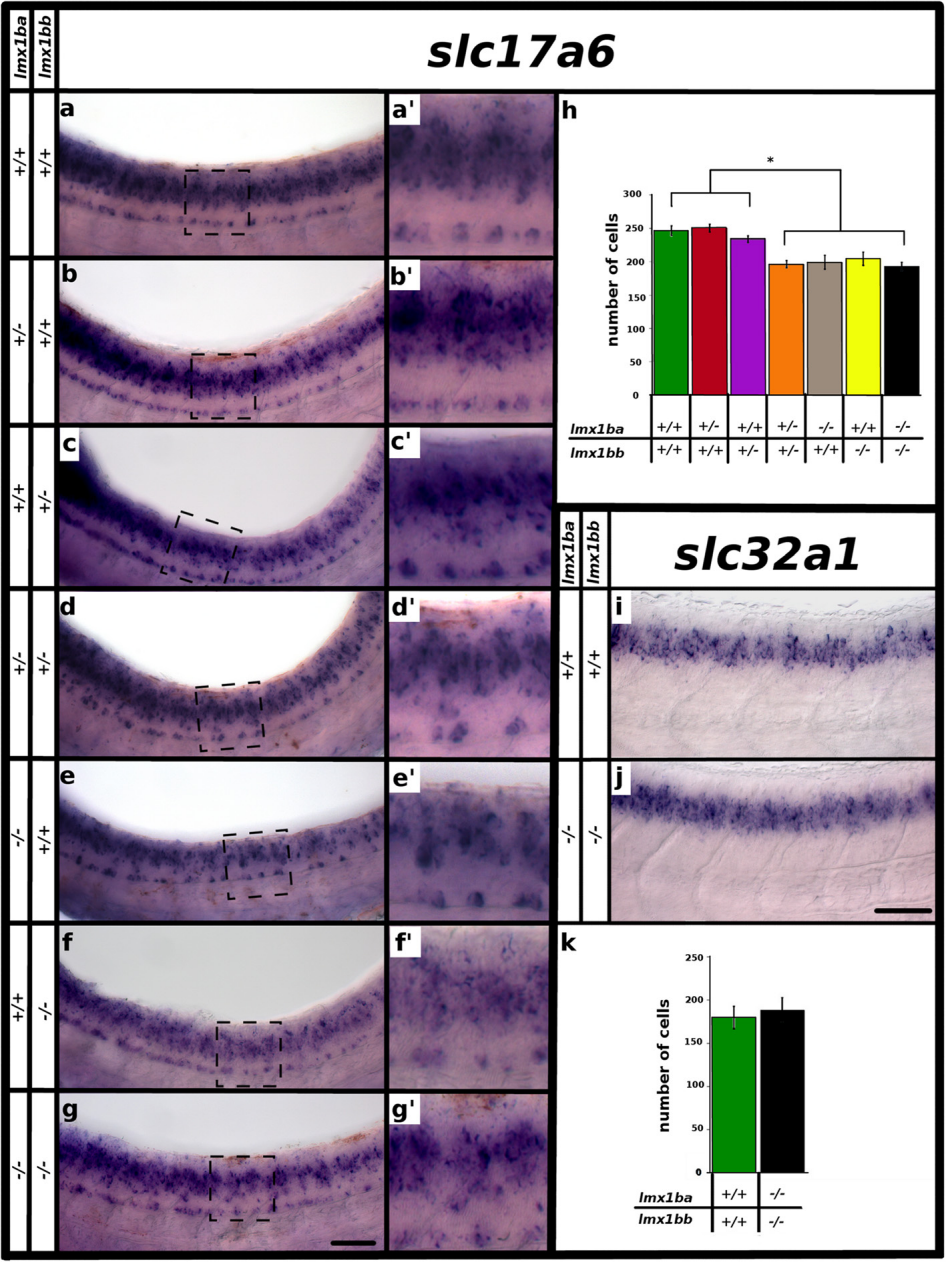
Three functional *lmx1b* alleles are required for correct numbers of glutamatergic cells at later developmental stages. Lateral view of zebrafish spinal cord at 48 h (**a**-**g’**, **i** and **j**), anterior left, dorsal top. *in situ* hybridization for *slc17a6a* + *slc17a6b* (*slc17a6*) (**a**-**g’**) and *slc32a1* (**i** and **j**). (**a’**-**g’**) are magnified views of black dashed box regions in panels (**a**-**g**). Columns on left indicate *lmx1ba* and *lmx1bb* genotype. Mean number of cells (y-axis) expressing *slc17a6* (**h**) and *slc32a1* (**k**) in spinal cord region adjacent to somites 6–10 at 48 h (x-axis). Square brackets and star in (**h**) indicates that each of the first three columns is statistically significantly different from each of the last four columns (*p* < 0.05). Embryo genotype is indicated below graph. Error bars indicate standard error of the mean. Two independent experiments were conducted for (**a**-**g**). Cell count results were similar in each replicate. One experiment was conducted for (**i** and **j**). Cell count data presented here (**h** and **k**) are average values of 4–13 embryos. Precise numbers of embryos counted and *p* values are provided in Table 5. Scale bar (**g**) = 50 μm (**a**-**g**) and 20 μm (**a’**-**g’**) and scale bar (**j**) = 50 μm (**i**, **j**)

When we examined *lmx1ba* single mutants at 48 h, we found that the number of glutamatergic neurons were statistically significantly reduced (*p* < 0.001) compared to WT siblings (Fig. 4e, e’ and h; Table 5). Interestingly, the number of glutamatergic neurons lost in the *lmx1ba* mutant was not statistically significantly different from the number of glutamatergic neurons lost in the *lmx1bb* mutant (*p* = 0.7; Fig. 4f, f’ and h; Table 5). More surprisingly, we also found that the number of spinal cord glutamatergic neurons lost in *lmx1ba;lmx1bb* double mutants, was not statistically significantly different from either *lmx1ba* single mutants (*p* = 0.78) or *lmx1bb* single mutants (*p* = 0.45; Fig. 4g, g’ and h; Table 5).

**Table 5:** *lmx1ba* and *lmx1bb* mutant alleles act redundantly in a dose-dependent manner

	48 h	*lmx1ba* ^*+/+*^ *lmx1bb* ^*+/+*^	*lmx1ba* ^*+/-*^ *lmx1bb* ^*+/+*^	*lmx1ba* ^*+/+*^ *lmx1bb* ^*+/-*^	*lmx1ba* ^*+/-*^ *lmx1bb* ^*+/-*^	*lmx1ba* ^*-/-*^ *lmx1bb* ^*+/+*^	*lmx1ba* ^*+/+*^ *lmx1bb* ^*-/-*^	*lmx1ba* ^*-/-*^ *lmx1bb* ^*-/-*^
*slc17a6*	Mean	246.3	250.2	233.8	196.4	199.4	204.5	193.1
	SEM	7.4	5.9	4.5	5.5	10.4	10	6.2
	n	8	5	4	8	11	9	13
	*p* value 1	n/a	0.72	0.3	**0.001**	**0.001**	**0.003**	**0.001**
	*p* value 2	0.3	0.08	n/a	**0.005**	**0.03**	**0.05**	**0.004**
	*p* value 3	**0.001**	**0.001**	**0.005**	n/a	0.66	0.38	0.78
	*p* value 4	**0.003**	**0.004**	**0.05**	0.38	0.7	n/a	0.45
	*p* value 5	**0.001**	**0.001**	**0.03**	0.66	n/a	0.7	0.78
*slc32a1*	Mean	179.8						188.5
	SEM	12.9						14.1
	n	6						6
	*p* value 1	n/a						0.94

Mean number of *slc17a6a* + *slc17a6b* (*slc17a6*) or *slc32a1-*expressing cells detected in the spinal cord region adjacent to somites 6–10 in 48 h embryos. SEM is the standard error of the mean. n is the number of embryos analyzed for each data set. *p* values are from student’s paired t-test. Statistically significant *p* values are indicated in bold. *p* value 1 is from comparing WT (*lmx1ba* ^*+/+*^ *;lmx1bb* ^*+/+*^) embryos pairwise with all other genotypes, *p* value 2 is from comparing *lmx1ba* ^*+/+*^ *;lmx1bb* ^*+/-*^ embryos pairwise with all other genotypes, *p* value 3 is from comparing *lmx1ba* ^*+/-*^ *;lmx1bb* ^*+/-*^ embryos pairwise with all other genotypes, *p* value 4 is from comparing *lmx1ba* ^*+/+*^ *;lmx1bb* ^*-/-*^ embryos pairwise with all other genotypes and *p* value 5 is from comparing *lmx1ba* ^*-/-*^ *; lmx1bb* ^*+/+*^ embryos pairwise with all other genotypes

Given the similarity of the phenotypes in *lmx1ba* and *lmx1bb* single and double mutants, we tested whether *lmx1bb* is required for *lmx1ba* spinal cord expression or vice versa. However, when we analyzed expression of *lmx1ba* in *lmx1bb* mutants and expression of *lmx1bb* in *lmx1ba* mutants we saw no obvious differences between WT and mutant embryos (Fig. 2c and e). This suggests that the phenotypic similarities between the mutants are not due to cross-regulation of these two *lmx1b* genes.

Together, these results suggest that *lmx1ba* and *lmx1bb* function partially redundantly in the spinal cord and that the presence of two or more mutant alleles (regardless of whether the mutation is in *lmx1ba* or *lmx1bb*) is sufficient to cause a reduction in the number of glutamatergic cells in the spinal cord. To test this, we examined the number of glutamatergic spinal neurons in *lmx1ba;lmx1bb* double heterozygous embryos and both *lmx1ba* and *lmx1bb* single heterozygous embryos. Consistent with our hypothesis, the reduction in the number of glutamatergic neurons in *lmx1ba;lmx1bb* double heterozygous embryos was not statistically significantly different from the reduction in *lmx1ba* mutants (*p* = 0.66), *lmx1bb* mutants (*p* = 0.38) or *lmx1ba;lmx1bb* double mutants (*p* = 0.78; Fig. 4d, d’ and h; Table 5). In contrast, neither *lmx1ba* nor *lmx1bb* single heterozygous embryos had a statistically significant reduction in the number of glutamatergic neurons when compared to WT siblings (*p* = 0.72 and *p* = 0.3 respectively; Fig. 4b-c’ and h; Table 5).

To test the possibility that *lmx1ba* might compensate for the loss of *lmx1bb* in the repression of inhibitory neurotransmitter phenotypes, we also analyzed the expression of *slc32a1* in *lmx1ba;lmx1bb* double mutants. However, like the *lmx1bb* single mutant results, the *lmx1ba;lmx1bb* double mutants had no statistically significant change (*p* = 0.94) in the number of spinal inhibitory neurons (Fig. 4i-k; Table 5). These data suggest that *lmx1ba* and *lmx1bb* are not required to repress (or specify) inhibitory neurotransmitter phenotypes and that the reduction in spinal cord glutamatergic cells in these mutants does not correlate with an increase in inhibitory cells.

### The reduction in spinal glutamatergic cells is not due to cell death

To test whether the reduction in glutamatergic neurons might be an indirect consequence of increased cell death, we used both acridine orange (AO) and an activated caspase-3 antibody [76, 77]. As the glutamatergic phenotype is comparable among *lmx1ba;lmx1bb* double mutants and both single mutants, we used *lmx1bb* single mutants for these and all subsequent experiments.

**Fig. 5:**
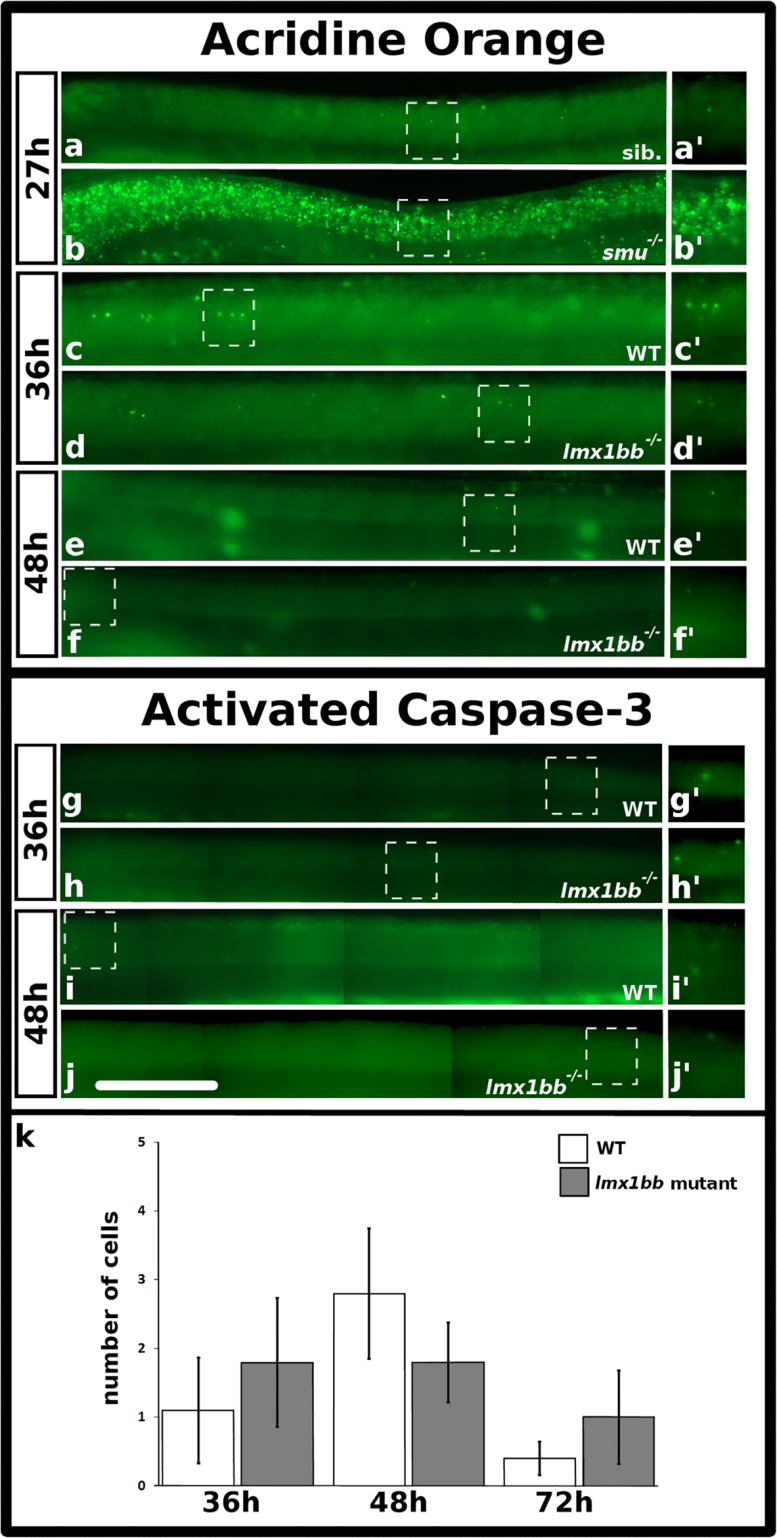
There is no increase in apoptosis in *lmx1bb* mutants between 36 h and 72 h. Lateral view of zebrafish spinal cord at 27 h (**a**-**b’**), 36 h (**c**-**d’** and **g**-**h’**) and 48 h (**e**-**f’** and **i**-**j’**), anterior left, dorsal top. Acridine orange (AO) treatment (**a**-**f’**) and activated caspase-3 immunohistochemistry as anterior-posterior montages (**g**-**j’**). Sib. in (**a**) is a sibling embryo to *smoothened* mutant in (**b**). (**a’**-**j’**) are magnified view of corresponding boxed region. Mean number of cells (y-axis) with activated caspase-3 staining in WT embryos (white) and *lmx1bb* homozygous mutants (grey) (x-axis) at indicated developmental times. Error bars indicate standard error of the mean. Two independent experiments were conducted for (**c**-**j**). Expression (**a**-**j**) and cell count data (**k**) were similar in each replicate. Data presented in (**k**) are average values of 5-8 embryos from the same experiment. Precise number of embryos counted and *p* values are provided in Table 6. Scale bar = 100 μm (**a**-**j**) and 80 μm (**a’**-**j’**)

AO is a vital dye that labels apoptotic cells in live zebrafish embryos [76, 78–81], as demonstrated in our positive control, *smoothened* mutant embryos, where many cells undergo apoptosis [76] (Fig. 5a-b’). We performed AO staining in *lmx1bb* mutants at 36 h, when we first observe a reduction in the number of glutamatergic spinal cells, and at 48 h, when the loss of glutamatergic spinal cord cells is more pronounced. At both of these time points there were no obvious differences in spinal cord AO staining in any of the live embryos derived from incrosses of heterozygous *lmx1bb* mutants (Fig. 5c-f’). Following imaging and analysis, a subset of embryos were genotyped to confirm that we had analyzed both WT and *lmx1bb* homozygous mutant embryos. These analyses demonstrated that there was no apparent correlation between the amount of AO staining and embryo genotype.

To confirm these results, we also assayed cell death using an activated caspase-3 antibody that has previously been used to successfully identify dying cells in zebrafish [82–84]. Activated caspase-3 immunohistochemistry was performed on embryos at 36 h, which is the first time point we detected a reduction of glutamatergic neurons, 48 h, when there is a larger reduction, and 72 h, which is 36 h after we first detected a reduction of glutamatergic neurons. At all of these stages we found no statistically significant difference in the number of activated caspase-3 cells when comparing WT and *lmx1bb* mutant embryos (*p* = 0.63 at 36 h; *p* = 0.4 at 48 h; *p* = 0.46 at 72 h; Fig. 5g-k; Table 6). Taken together, these AO and activated caspase-3 experiments suggest that there is no increase in cell death in *lmx1bb* mutant spinal cords, at least between 36 and 72 h.

**Table 6:** Activated caspase-3 is not increased in zebrafish *lmx1bb* mutants during the first 72 h of development

		36 h		48 h		72 h	
Marker		WT	*lmx1bb* ^*-/-*^	WT	*lmx1bb* ^*-/-*^	WT	*lmx1bb* ^*-/-*^
Activated Caspase-3	Mean	1.1	1.8	2.8	1.8	0.4	1
	SEM	0.77	0.94	0.95	0.58	0.24	0.68
	n	8	7	6	5	6	5
	*p* value	0.63		0.4		0.46	

Mean number of activated caspase-3-expressing cells at 36, 48 and 72 h within the entire spinal cord. SEM is the standard error of the mean. n is the number of embryos analyzed for each experiment. *p* value is from a student’s paired t-test comparing WT embryos and *lmx1bb* homozygous mutants

### V0v and dI5 cells form in normal numbers in *lmx1bb* mutants

**Fig. 6:**
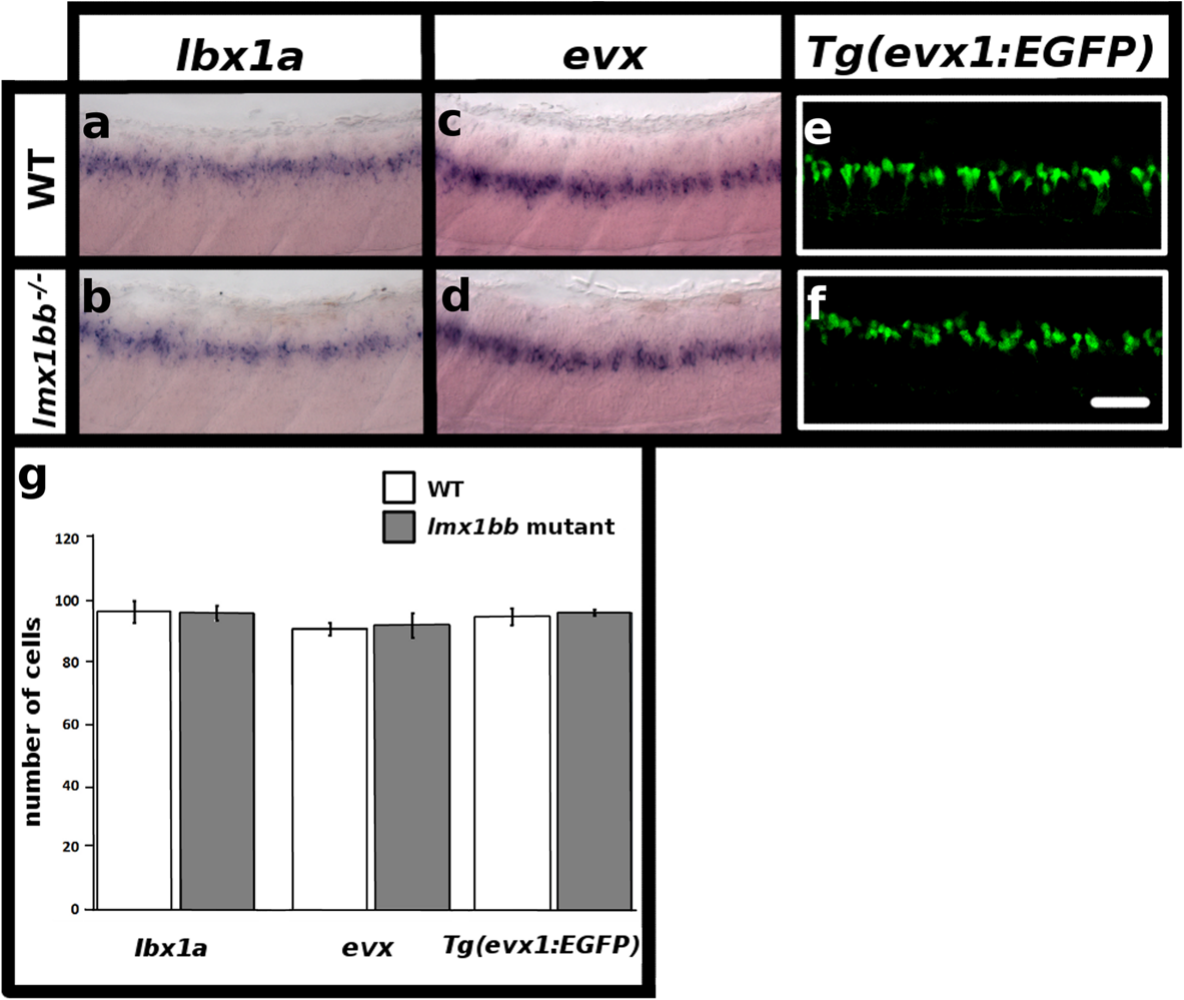
*lmx1bb* is expressed by dI5 and V0v neurons and these cells form in normal numbers in *lmx1bb* mutants. Lateral view of zebrafish spinal cord at 48 h (**a-f**), anterior left, dorsal top. *in situ* hybridization for *lbx1a* in WT (**a**) and *lmx1bb* homozygous mutant (**b**) embryos. *in situ* hybridization for *evx1 + evx2* (*evx*) in WT (**c**) and *lmx1bb* homozygous mutant (**d**) embryos. Immunohistochemistry for EGFP in *Tg(evx1:EGFP)* ^*SU1*^ WT (**e**) and *lmx1bb* homozygous mutant (**f**) embryos. **g** Mean number of cells (y-axis) in WT embryos (white) and *lmx1bb* homozygous mutants (grey) expressing *lbx1a, evx* or *Tg(evx1:EGFP)* ^*SU1*^ in spinal cord region adjacent to somites 6–10 (x-axis). Two independent experiments were conducted for (**a-f**). Cell count results were similar in each replicate. Data shown here (**g**) are average values of 6–10 embryos from the same experiment. Precise number of embryos counted and *p* values are provided in Table 4. Error bars indicate standard error of the mean. Scale bar = 50 μm (**a-f**)

Since *lmx1bb* is co-expressed by both *lbx1a-*expressing dI5 cells and *evx-*expressing V0v neurons (Fig. 1h and i), we also examined whether *lbx1a* or *evx1* + *evx2* (a mixture of probes for both genes, referred to here as *evx*) expression was altered in *lmx1bb* mutants. At 48 h there was no statistically significant difference in the number of cells expressing *lbx1a* or *evx* in *lmx1bb* mutants compared to WT siblings (*p* = 0.93 and *p* = 0.78 respectively; Fig. 6a-d and g; Table 4). Additionally, there was no statistically significant change in the number of EGFP-labeled V0v neurons in *lmx1bb* mutants expressing the *Tg(evx1:EGFP)*^*SU1*^ transgene when compared to WT siblings (*p* = 0.63; Fig. 6e-g; Table 4). These data suggest that Lmx1bb function is not required for either *lbx1a* or *evx* expression and that V0v and dI5 cells form in normal numbers in *lmx1bb* mutants. This is consistent with our previously described apoptosis assays which suggest that spinal neurons are not dying in these mutants (Fig. 5). Furthermore, these findings suggest that there is no effect on V0v or dI5 cell proliferation and that these cells are not transfating into different cell types in the *lmx1bb* mutants, they are just losing or do not develop their glutamatergic fates.

### *lmx1bb* is required for the glutamatergic phenotype of at least a subset of V0v interneurons

**Fig. 7:**
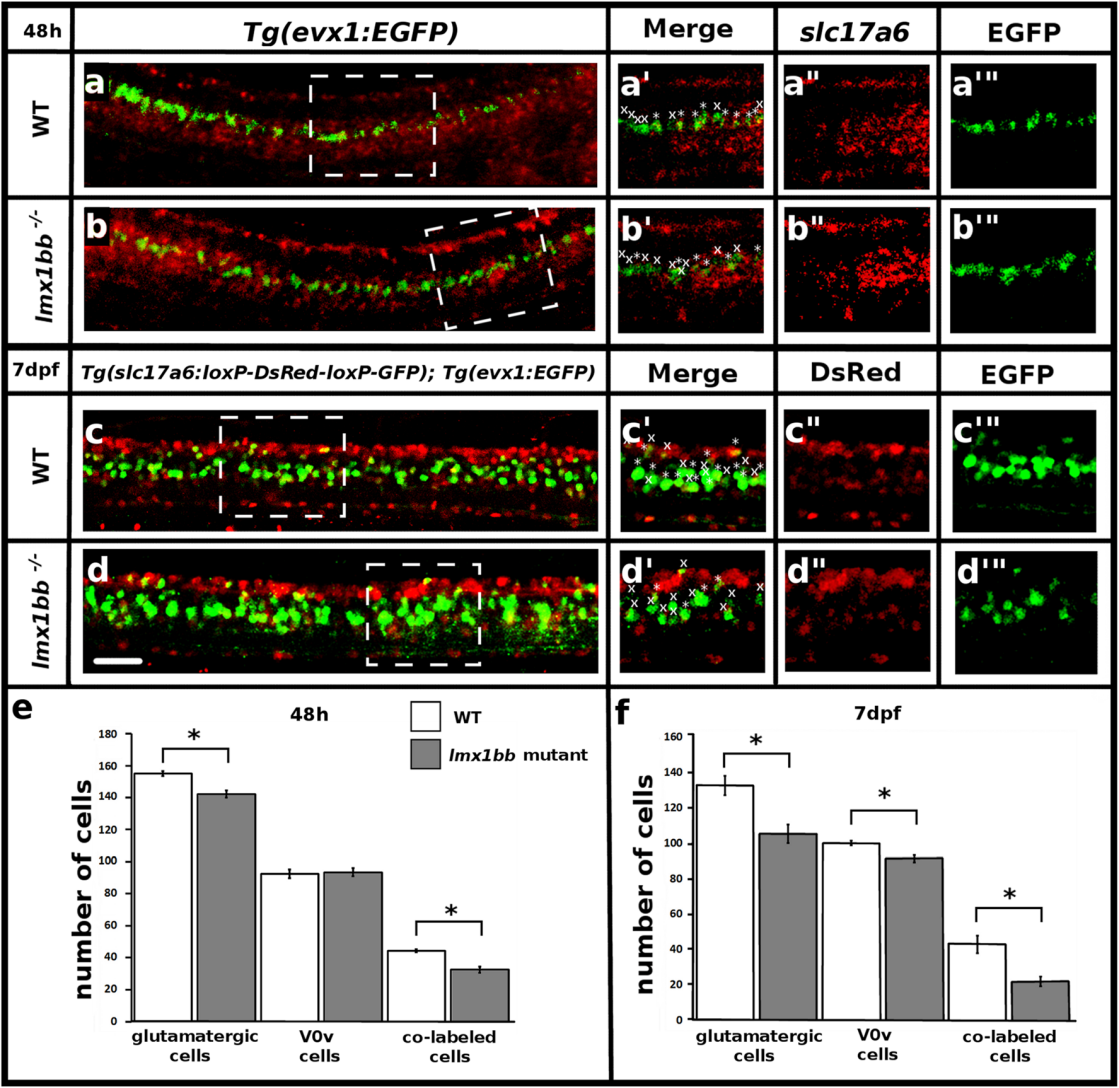
*lmx1bb* is required for V0v interneuron glutamatergic fates at later stages of development. Lateral view of zebrafish spinal cord at 48 h (**a** and **b**) and 7 dpf (**c** and **d**), anterior *left*, dorsal top. *in situ* hybridization *slc17a6* (red) and EGFP immunohistochemistry (green) in WT (**a**) and *lmx1bb* mutant (**b**) *Tg(evx1:EGFP)* ^*SU1*^ embryos. Single magnified confocal plane from white dashed box region (**a’**-**a’”** and **b’**-**b’”**). Immunohistochemistry for EGFP (green) and DsRed (red) in WT (**c**) and *lmx1bb* mutant (**d**) *Tg(slc17a6b(vglut2a):loxP-DsRed-loxP-GFP)* ^*nns14*^ *;Tg(evx1:EGFP)* ^*SU1*^ embryos. Single magnified confocal plane from white dashed box region (**c’**-**c’”** and **d’**-**d’”**). * indicates co-labeled cell, x indicates single labeled EGFP-expressing (V0v) cell. (**e** and **f**) Mean number of cells (y-axis) expressing *slc17a6* or DsRed (glutamatergic), EGFP (V0v) and *slc17a6* or DsRed + EGFP (co-labeled) (x-axis) in WT (white) and *lmx1bb* homozygous mutants (grey). Error bars indicate standard error of the mean. Three independent experiments were conducted for (**c** and **d**). Cell count results were similar in each replicate. One experiment was conducted for (**a** and **b**). Data shown here (**e** and **f**) are average values of 5–12 embryos. Precise number of embryos counted and p values are provided in Table 7. The glutamatergic and V0v numbers include co-labeled cells. Statistically significant (*p* < 0.05) comparisons are indicated with square brackets and stars. Scale bar = 30 μm (**a**-**d**) and 25 μm (**a’**-**d’”**)

As described above (Fig. 1i), we have shown for the first time in any animal that at least a subset of V0v neurons express *lmx1bb*. To test whether V0v cells in particular are affected in *lmx1bb* mutants we performed double labels for EGFP and *slc17a6* in WT and *lmx1bb* mutant *Tg(evx1:EGFP)*^*SU1*^ embryos. We found that at 48 h, there is a significant reduction in the number of glutamatergic double-labeled V0v cells in mutant embryos compared to their WT siblings (*p* < 0.001; Fig. 7a, b and e; Table 7). This suggests that at least some of the cells that are losing their glutamatergic phenotypes in *lmx1bb* mutants are V0v neurons.

**Table 7:** Lmx1b regulates the glutamatergic phenotype of a subset of V0v neurons

	*slc17a6* (glutamatergic)		EGFP (V0v)		co-labeled	
48 h	WT	*lmx1bb* ^*-/-*^	WT	*lmx1bb* ^*-/-*^	WT	*lmx1bb* ^*-/-*^
Mean	155	142.2	92.3	93.4	44.3	32.4
SEM	1.54	2.22	2.72	2.58	0.9	1.9
n	6	5	6	5	6	5
*p value*	**<0.001**		0.76		**<0.001**	
	DsRed (glutamatergic)		EGFP (V0v)		co-labeled	
7 dpf	WT	*lmx1bb* ^*-/-*^	WT	*lmx1bb* ^*-/-*^	WT	*lmx1bb* ^*-/-*^
Mean	132.6	105.3	100.1	91.3	42.9	21.8
SEM	5.5	5.2	1.4	2.1	4.9	2.7
n	9	12	9	12	9	12
*p* value	**<0.005**		**0.002**		**<0.001**	

Mean number of cells expressing *slc17a6*, EGFP or both in the spinal cord region adjacent to somites 6–10 in 48 h embryos and the mean number of cells expressing DsRed, EGFP or both in the spinal cord region adjacent to somites 6–10 in 7 dpf embryos. SEM is the standard error of the mean. n is the number of embryos analyzed for each data set. *p* value is from a student’s paired t-test comparing WT embryos and *lmx1bb* homozygous mutants. Statistically significant values are indicated in bold

We were also interested in establishing if the reduction of glutamatergic cells in general and/or the reduction in the number of glutamatergic V0v cells persists at later stages of development. As our *slc17a6* RNA probe does not label cells effectively in double staining experiments at later stages of development, we created fish transgenic for both *Tg(slc17a6b:loxP-DsRed-loxP-GFP)*^*nns14*^ and *Tg(evx1:EGFP)*^*SU1*^ and heterozygous for the *lmx1bb* mutation. Embryos from these parents express DsRed in glutamatergic neurons and EGFP in V0v neurons [11, 85, 86]. We then counted single and double-labeled cells in WT and *lmx1bb* mutant embryos at 7 dpf to determine if the number of excitatory cells and excitatory V0v neurons in particular were reduced in the *lmx1bb* mutants. We again observed a statistically significant (*p* < 0.005) reduction in the total number of glutamatergic, DsRed-labeled neurons (Fig. 7d and f; Table 7) as well as a statistically significant reduction in the number of glutamatergic DsRed-labeled V0v neurons (*p* < 0.001; Fig. 7d and f; Table 7) in *lmx1bb* mutant embryos compared to WT siblings. More surprisingly, we also observed a very small but statistically significant (*p* = 0.002) reduction in the number of V0v (EGFP-labeled) neurons at 7 dpf in *lmx1bb* mutants (Fig. 7d’” and f; Table 7). However, this slight reduction was substantially less than the reduction in double-labeled glutamatergic V0v neurons, demonstrating that the second result cannot be explained by the first. Taken together, these results suggest that *lmx1bb* is required for the glutamatergic neurotransmitter phenotype of at least a subset of V0v neurons at later developmental stages.

### *lmx1ba* and *lmx1bb* expression requires *evx1* and *evx2*

**Fig. 8:**
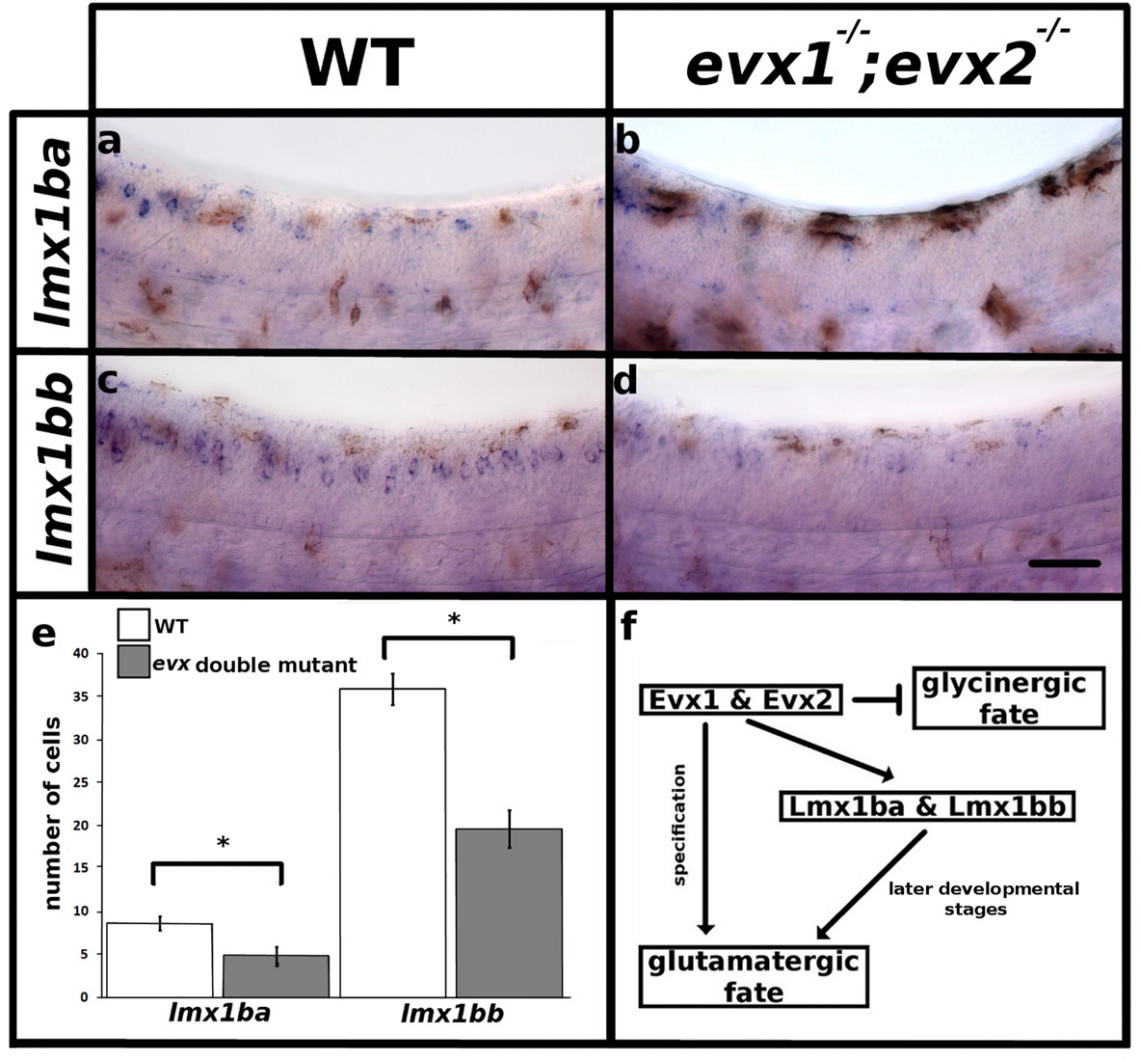
Evx1 and Evx2 are required for *lmx1b* expression. Lateral view of zebrafish spinal cord at 30 h (**a**-**d**), anterior, left dorsal top. *in situ* hybridization for *lmx1ba* (**a**, **b**) and *lmx1bb* (**c**, **d**). Brown coloration in (**a**-**d**) is pigment from melanocytes. **e** Mean number of cells (y-axis) that express *lmx1ba* and *lmx1bb* in WT (white) and *evx1;evx2* double mutants (grey) (x-axis). Error bars indicate standard error of the mean. Statistically significant (*p* < 0.05) comparisons are indicated with square brackets and stars. One experiment was conducted for (**a** and **b**). Two independent experiments were conducted for (**c** and **d**) and cell count results were similar in each replicate. Data presented here (**e**) are average values for 5–12 embryos. Precise number of embryos counted and *p* values are provided in Table 8. **f**. Proposed mechanism for how the excitatory (glutamatergic) neurotransmitter phenotype of at least a subset of V0v neurons is specified and/or maintained. We previously demonstrated that Evx1 & Evx2 specifies the excitatory (glutamatergic) neurotransmitter phenotype and represses inhibitory (glycinergic) phenotypes in V0v cells [11]. The current study demonstrates that Evx1 & Evx2 are also required for *lmx1ba* and *lmx1bb* expression. Furthermore, we show that Lmx1bb is required at later developmental stages either to maintain the excitatory (glutamatergic) neurotransmitter phenotype for at least a subset of V0v neurons or to specify the glutamatergic phenotype of a later-forming subset of V0v cells. Scale bar = 50 μm

Evx1 and Evx2 function partially redundantly to specify the glutamatergic neurotransmitter phenotype of V0v neurons [11]. As demonstrated above (Fig. 7), *lmx1bb* is required at later developmental stages for the glutamatergic neurotransmitter phenotype of at least a subset of V0v neurons and *evx1* and *evx2* spinal cord expression is normal in *lmx1bb* mutants (Fig. 6c-g), suggesting that Evx1 and Evx2 do not act downstream of Lmx1b in V0v cells. To determine whether Lmx1b acts downstream of Evx1 and Evx2 or in a parallel pathway we examined *lmx1b* expression in *evx1;evx2* double mutants. At 30 h we found that there was a statistically significant reduction in the number of *lmx1ba* (*p* = 0.029) and *lmx1bb* (*p* < 0.001) expressing spinal cord cells in *evx1;evx2* double mutants when compared to WT siblings (Fig. 8a-e; Table 8). This suggests that these two genes require Evx function for their expression in V0v cells and that they are downstream of *evx1* and *evx2* in these cells (Fig. 8f).

**Table 8:** Evx1 and Evx2 are required for *lmx1b* expression

Marker	30 h	WT	*evx1* ^*-/-*^ *;evx2* ^*-/-*^
*lmx1ba*	Mean	8.6	4.8
	SEM	0.81	1.1
	n	5	6
	*p* value	**0.029**	
*lmx1bb*	Mean	35.9	19.7
	SEM	6.4	5.3
	n	12	6
	*p* value	**<0.001**	

Mean number of *lmx1ba* or *lmx1bb-*expressing cells in the spinal cord region adjacent to somites 6–10 in 30 h embryos. SEM is the standard error of the mean. n is the number of embryos analyzed for each data set. *p* value is from a student’s paired t-test comparing WT and *lmx1bb* mutant embryos. Statistically significant *p* values are indicated in bold

## Discussion

In this paper we demonstrate that *lmx1ba* and *lmx1bb* (zebrafish ohnologs of *Lmx1b*) are both expressed by spinal cord interneurons during development (Figs. 1, 2 and 8). This is consistent with a previous report where *lmx1bb* was shown to be expressed by anterior spinal neurons [24], but in contrast, this earlier study suggested that *lmx1ba* was not expressed in the zebrafish spinal cord [24]. Given that we have only detected very weak spinal cord staining with our *in situ* hybridizations for *lmx1ba*, despite trying three different RNA *in situ* probes (see methods), we think that the spinal cord staining of *lmx1ba* was too weak to be easily detected in these previous experiments.

Consistent with results in mouse [16, 18, 32, 62–64, 87], we demonstrate here that in the zebrafish spinal cord *lmx1bb* is predominantly expressed by glutamatergic neurons (Fig. 1j and k). Our results demonstrate that at least 79 % of *lmx1bb-*expressing neurons are glutamatergic (Fig. 1j and k; Table 2). We consistently see fewer labeled cells with the *Tg*(*slc17a6:EGFP)* and *Tg(slc17a6b:loxP-DsRed-loxP-GFP)*^*nns14*^ lines than we do with single *in situ* hybridization for *slc17a6* (Fig. 1j and k; Table 2; data not shown), suggesting these transgenic lines either do not label all spinal cord glutamatergic cells at the stages examined or there is a delay in the expression of the fluorescent proteins compared to *slc17a6* RNA. *slc17a6* is also not a strong probe in double-labeling experiments and as a result it labels slightly fewer cells in double *in situ* hybridizations than in single *in situ* hybridizations. This suggests that some of the *lmx1bb*-positive, *slc17a6*-negative cells in our double labels may also be glutamatergic. Therefore, it is possible that more than 79 % of *lmx1bb-*expressing neurons are glutamatergic, especially as we also show that only about 10 % of *lmx1bb*-expressing cells are inhibitory (Fig. 1l; Table 2).

Also consistent with results in amniotes [16, 18, 32, 62–64, 87], our analyses suggest that a subset of *lmx1b-*expressing spinal cord cells are dI5 cells. dI5 cells constitute about a third of all *Lbx1-*expressing spinal cord cells and they are also the only excitatory, *Lbx1*-expressing spinal cells [8–10, 16]. We find that at least 45 % of *lmx1bb*-expressing spinal cells co-express *lbx1a* and that these co-expressing cells constitute about a third of the *lbx1a*-expressing cells (Fig. 1h; Table 2). Together with the fact that most *lmx1bb-*expressing cells are glutamatergic, this suggests that at least most of the cells that co-express *lmx1bb* and *lbx1a* are dI5 cells.

However, in contrast to previous reports in amniotes, we also find that a substantial proportion of *lmx1bb*-expressing spinal cells (at least 38 %) are V0v neurons (Fig. 1i; Table 2). This is the first time that *lmx1bb* expression has been described in this cell type in any animal. However, a small subset of Lmx1b cells are located in the ventral spinal cord of E12.5 mice [32] in a region similar to where Evx1, a V0v marker, is expressed [12]. Therefore, it is possible that some mouse V0v neurons may also express Lmx1b. Interestingly, at the stages that we examined, only a subset of zebrafish V0v neurons express *lmx1bb* (Fig. 1i; Table 2). This suggests that *lmx1bb* may be expressed by a specific subset of V0v interneurons. Interestingly, Satou and colleagues have shown that V0v neurons can be divided into three subsets based on their morphology [31]. Alternatively, it is possible that all V0v cells express *lmx1bb*, but either only transiently, or only at later stages of their development, resulting in only a subset being co-labeled at any particular time.

While we were unable to successfully perform double-labeling experiments with *lmx1ba* and *lmx1bb*, due to very weak staining with our *lmx1ba* RNA probes, our results suggest that these two genes are expressed by the same spinal cord neurons. Firstly, their spinal cord expression patterns are very similar, although *lmx1ba* is expressed later than *lmx1bb* in most spinal cord domains (Figs. 1d-g, 2a-f and 8a and c). Secondly, *lmx1ba* single mutant, *lmx1bb* single mutant, *lmx1ba;lmx1bb* double mutant and *lmx1ba;lmx1bb* double heterozygous embryos all have the exact same spinal cord phenotype (the same reduction in the number of glutamatergic neurons) suggesting that the two ohnologs have redundant functions in the spinal cord, and must, therefore, be co-expressed in at least some cells (Fig. 4; Table 5). Interestingly, other studies using zebrafish to examine *lmx1ba* and *lmx1bb* functions in the isthmus, diencephalon and eye have also found that these two genes have overlapping expression and function redundantly in those tissues [21, 23, 24]. Finally, in *evx1;evx2* double mutants the number of cells expressing either *lmx1ba* or *lmx1bb* is reduced (Fig. 8; Table 8). Given that *evx1* and *evx2* are only expressed in V0v neurons in the spinal cord, this strongly suggests that both of the *lmx1b* ohnologs are expressed in V0v cells.

In this paper, we demonstrate that Lmx1b transcription factors are required in zebrafish for correct numbers of spinal glutamatergic cells at later stages of development. At 27 h there is no change in the number of spinal glutamatergic cells in the spinal cord in *lmx1bb* mutants, but by 36 h there is a reduction in the number of glutamatergic cells and this reduction becomes more severe by 48 h. We also demonstrate that this phenotype persists until at least 7 days. In contrast, *lmx1b*-expressing dI5 and V0v cells are still present in normal numbers at 48 h and we observe no increase in cell death at either 36 h or 48 h. These results suggest that the reduction of glutamatergic cells is not a consequence of cell death, changes in cell proliferation or global changes in cell fate.

Prior to this study, *Lmx1b* had been implicated in correct neuronal migration, connectivity and viability [18, 19, 25, 62, 88, 89]. However, while data from previous studies suggested that *Lmx1b* may have a role in the development of a subset of spinal cord glutamatergic cells (e.g. [8, 9, 18, 62]), a precise function in glutamatergic fate specification or maintenance had not been identified. Interestingly, when *Lmx1b* was conditionally ablated specifically in the mouse spinal cord, at E18.5 there was also a reduction in the number of glutamatergic neurons and no change in the number of inhibitory neurons, which is similar to our results in zebrafish (Figs. 3 and 4) [18]. However, the authors of this study attributed this reduction in glutamatergic neurons to cell death because they also observed a reduction in the total number of cells in the dorsal horn and an increase in caspase-3-positive neurons. Despite this, the authors speculated that Lmx1b may function in the maintenance of the neurotransmitter phenotype prior to the death of these cells [18].

Our results differ from this mouse study because we do not see any evidence of an increase in cell death in the spinal cord of zebrafish *lmx1bb* mutants, at least between 36 and 72 h, even though we see statistically significant reductions in spinal glutamatergic cells at these stages. It is possible that the slight reduction in EGFP-labeled V0v cells that we see at 7 dpf might be due to cell death, but if this is the case this is likely to be a second, later phenotype as it occurs much later than the reduction in glutamatergic cells and affects a much smaller number of cells than the glutamatergic phenotype. Future studies could perform cell death assays to test whether V0v cells die at later stages, but this would not be trivial as this death could occur any time between 72 h and 7 dpf and the number of cells lost at 7 dpf is very small.

The lack of cell death in zebrafish *lmx1bb* mutants at earlier developmental stages where we see neurotransmitter phenotypes might seem surprising given the evidence that mis-programmed cells die in the mammalian spinal cord [18, 88, 89]. This result could possibly be the consequence of different developmental strategies being utilized in mouse and zebrafish spinal cords. In zebrafish embryos, with the exception of Rohon Beard cells, there is very little apoptosis in the spinal cord during development [90], compared to substantially more programed cell death in the mouse spinal cord [91–93]. It is possible that the fast development and/or smaller size of zebrafish embryos makes it more difficult to utilize a strategy of creating and pruning excess neurons. In this case, mice might be better equipped to eliminate neurons with incorrect functional characteristics than zebrafish. Consistent with this hypothesis, in an earlier study when we removed Pax2 and Pax8 function in zebrafish, several subsets of spinal interneurons lost their inhibitory neurotransmitter phenotypes but they were still present in normal numbers and had normal morphologies and axonal trajectories [7], suggesting that their viability was not affected. Similarly, in zebrafish *evx1;evx2* double mutants, V0v spinal neurons switch their neurotransmitter phenotypes from excitatory to inhibitory but they retain normal axon projections until at least 48 h, again suggesting that their viability is not affected [11].

Regardless of the reason, we do not see any evidence of increased cell death in the spinal cord of zebrafish *lmx1bb* mutants between 36 and 72 h. We also do not see any change in the numbers of V0v or dI5 cells, suggesting that the cells that usually express *lmx1bb* still form and are present in normal numbers (Fig. 6; Table 4). However, in contrast and as discussed above, our data suggest that Lmx1b transcription factors are required either to maintain the glutamatergic neurotransmitter phenotype of a subset of excitatory spinal neurons or to specify the glutamatergic phenotype of a later-forming subset of spinal neurons (Figs. 3 and 4). If Lmx1bb is required to maintain a subset of glutamatergic fates in the spinal cord, this would be consistent with Lmx1b function in some other regions of the CNS, specifically the mouse raphe nucleus and trigeminal brainstem complex, where Lmx1b is required to maintain specific neurotransmitter phenotypes [19, 25, 94, 95]. However, in the case of the raphe nucleus it is a serotonergic phenotype rather than a glutamatergic phenotype that Lmx1b maintains.

As *lmx1bb* has not previously been shown to be expressed by V0v neurons, we were particularly interested in testing whether *lmx1bb* is specifically required for the glutamatergic phenotypes of these cells. We found that there is a statistically significant reduction in the number of glutamatergic V0v cells in *lmx1bb* mutants at both 48 h and 7dpf (Fig. 7; Table 7). Despite the fact that previous reports have shown that all V0v cells are excitatory [11, 31], not all WT V0v neurons were co-labeled with *slc17a6* or *Tg(slc17a6b(vglut2a):loxP-DsRed-loxP-GFP*)^*nns14*^ in these experiments. This is probably because, as discussed above, *slc17a6* is a weak probe in double labelling experiments and the *Tg(slc17a6b(vglut2a):loxP-DsRed-loxP-GFP)*^*nns14*^ transgenic line only labels a subset of glutamatergic spinal cord cells in our hands. In contrast, we observed the same number of *evx-*expressing cells by *in situ* hybridization as EGFP-positive cells in *Tg(evx1:EGFP)*^*SU1*^ embryos (Fig. 6c-g; Table 4), suggesting that this transgenic line labels all V0v cells, even at later stages of development, as has previously been shown for earlier stages [11].

In addition to showing that *lmx1bb* is expressed by V0v cells, our results also demonstrate that *lmx1ba* and *lmx1bb* expression in these cells requires Evx1 and Evx2 activity. When we examine *lmx1ba* and *lmx1bb* expression in *evx1;evx2* double mutant embryos we see a statistically significant reduction in the number of *lmx1ba* and *lmx1bb-*expressing neurons (Fig. 8a-e; Table 8). Our previous work demonstrated that Evx1 and Evx2 function partially redundantly to specify the glutamatergic neurotransmitter phenotype of V0v neurons [11]. Combined with these earlier results, the data in this paper start to elucidate a pathway of neurotransmitter fate specification and maintenance for V0v cells, with Evx1 and Evx2 specifying the glutamatergic neurotransmitter phenotype as well as *lmx1ba* and *lmx1bb* expression. The *lmx1b* genes then function downstream of Evx1 and Evx2, either to maintain the glutamatergic neurotransmitter phenotype of at least a subset of V0v neurons or to specify the glutamatergic neurotransmitter phenotype of a late-forming subset of V0v cells (Fig. 8f).

Interestingly, our results also show that correct Lmx1b function requires three functional *lmx1b* alleles in zebrafish, but it does not seem to matter which *lmx1b* alleles these are (Fig. 4). This suggests that *lmx1ba* and *lmx1bb* are at least partially redundant. Given that *lmx1ba* and *lmx1bb* are ohnologs that presumably arose in the teleost specific genome duplication event [96, 97], this requirement for three functional *lmx1b* alleles must have arisen in the teleost lineage. Interestingly, in humans, just one mutant allele of *Lmx1b* causes the autosomal dominant disorder nail-patella syndrome (NPS), suggesting that gene dosage is also important in mammals [98, 99]. Moreover, a quarter of NPS patients experience peripheral neurological symptoms which may be the result of improper specification of spinal cord neurons [18, 62, 100], suggesting that our results may have direct relevance to this human disorder.

During these studies we also discovered that *slc32a1,* previously believed to be expressed by all inhibitory neurons, does not label all inhibitory spinal neurons at 48 h in zebrafish [33, 68]. At 48 h *slc32a1* expression is restricted to a band of neurons in the middle of the dorsal-ventral axis of the spinal cord (Fig. 3l), whereas the GADs (markers of GABAergic cells) are also expressed in more ventral regions at this stage (Fig. 3l and o). At 27 h *slc32a1* seems to be expressed by all inhibitory neurons, as previously reported [33, 68]. However, at 36 h *slc32a1* expression starts to be restricted to more dorsal inhibitory populations, although some ventral *slc32a1-*expressing cells are still detected. It is this sporadic ventral *slc32a1* expression that likely causes the larger variations in the number of *slc32a1-*expressing cells detected at 36 h when compared to 27 h and 48 h (Fig. 3j and k; Table 3).

## Conclusions

In conclusion, we demonstrate that *lmx1ba* and *lmx1bb* are expressed by V0v and dI5 spinal interneurons. These genes are required, partially redundantly, in a dose-dependent manner, for the glutamatergic neurotransmitter phenotype of at least a subset of these neurons at later developmental stages. However, *lmx1ba* and *lmx1bb* are not required to repress (or specify) inhibitory neurotransmitter phenotypes as there is no statistically significant change in the number of inhibitory cells in either *lmx1ba* or *lmx1bb* single or double mutants. We also show that *lmx1ba* and *lmx1bb* require Evx1 and Evx2 for their expression in V0v neurons, suggesting that *lmx1ba* and *lmx1bb* act downstream of Evx1 and Evx2 in specifying or maintaining the glutamatergic neurotransmitter phenotype of at least a subset of V0v neurons. Taken together, our results provide new and powerful insights into the mechanisms required for excitatory neurotransmitter phenotypes within the spinal cord.

## Abbreviations

AO: Acridine Orange
CNS: Central Nervous System
DABCO: 1,4-diazabicyclo[2.2.2]octane
DIC: Differential Interference Contrast
DMSO: Dimethyl Sulfoxide
dpf: Days Post Fertilization
FACS: Fluorescent Activated Cell Sorting
GADs: Glutamic Acid Decarboxylases
h: Hours Post Fertilization
IACUC: Institutional Animal Care and Use Committee
NPS: Nail-patella Syndrome
PBS: Phosphate-buffered Saline
PTU: 1-phenyl 2-thiourea
WT: Wild-type
